# Assessing cognition in people with severe mental disorders in low- and middle-income countries: a systematic review of assessment measures

**DOI:** 10.1007/s00127-021-02120-x

**Published:** 2021-06-18

**Authors:** Yohannes Gebreegziabhere Haile, Kassahun Habatmu, Andualem Derese, Hetta Gouse, Stephen M. Lawrie, Matteo Cella, Atalay Alem

**Affiliations:** 1 Department of Nursing, College of Health Sciences, Debre Berhan University, Debre Berhan, Ethiopia; 2 Department of Psychiatry, College of Health Sciences, Addis Ababa University, Addis Ababa, Ethiopia; 3 School of Psychology, College of Education and Behavioral Studies, Addis Ababa University, Addis Ababa, Ethiopia; 4 Department of Public Health, College of Health Sciences, Haremaya University, Harar, Ethiopia; 5 Department of Psychiatry and Mental Health, University of Cape Town, Cape Town, South Africa; 6 Department of Psychiatry, University of Edinburgh, Edinburgh, Scotland, UK; 7 Department of Psychology, Institute of Psychiatry, Psychology and Neuroscience, King’s College London, London, England, UK

**Keywords:** Cognition, Measures, Psychometric, Severe mental disorder

## Abstract

**Background:**

Cognitive difficulties are common in people with severe mental disorders (SMDs) and various measures of cognition are of proven validity. However, there is a lack of systematic evidence regarding the psychometric properties of these measures in low- and middle-income countries (LMICs).

**Objective:**

To systematically review the psychometric properties of cognitive measures validated in people with SMDs in LMICs.

**Methods:**

We conducted a systematic review of the literature by searching from four electronic databases. Two authors independently screened studies for their eligibility. Measurement properties of measures in all included studies were extracted. All eligible measures were assessed against criteria set for clinical and research recommendations. Results are summarized narratively and measures were grouped by measurement type and population.

**Results:**

We identified 23 unique measures from 28 studies. None of these was from low-income settings. Seventeen of the measures were performance-based. The majority (*n* = 16/23) of the measures were validated in people with schizophrenia. The most commonly reported measurement properties were: known group, convergent, and divergent validity (*n* = 25/28). For most psychometric property, studies of methodological qualities were found to be doubtful. Among measures evaluated in people with schizophrenia, Brief Assessment of Cognition in Schizophrenia, Cognitive Assessment Interview, MATRICS Consensus Cognitive Battery, and CogState Schizophrenia Battery were with the highest scores for clinical and research recommendation.

**Conclusions:**

Studies included in our review provide only limited quality evidence and future studies should consider adapting and validating measures using stronger designs and methods. Nonetheless, validated assessments of cognition could help in the management and allocating therapy in people with SMDs in LMICs.

**Supplementary Information:**

The online version contains supplementary material available at 10.1007/s00127-021-02120-x.

## Introduction

Severe mental disorders (SMDs) are defined as having a non-organic psychosis with long illness duration and severe functional impairment [1]. SMDs include schizophrenia, bipolar disorder, and major depressive disorder with psychotic features. Despite their relatively low prevalence, these disorders are among the leading causes for Years Lived with Disability (YLD) [2]. Research shows that people with SMDs have significantly more cognitive difficulties compared to healthy controls [3–7]. In support of this, a recent systematic review showed that cognitive symptoms in people with schizophrenia (PWS) had heterogenous trajectories [8].

Cognition is a term referring to thinking skills including acquiring and retaining knowledge, processing information, and reasoning. Cognitive function includes intellectual abilities such as perception, reasoning, and remembering. Impairment in those functions (i.e., memory, judgment, and comprehension) is referred to as cognitive impairment [9]. PWS tend to have greater cognitive impairment compared to people with bipolar disorder (PWBD) and people with depression (PWD) [10–13].

Even though SMDs share nearly similar domains of cognitive impairment, the impairment in PWS is more global compared to the impairment in PWBD and PWD. Both PWS and PWBD show impairment in the domains of attention, verbal learning, and executive function [14, 15]. Whereas, domains of processing speed, working memory, verbal and visual learning, and reasoning are impaired in PWS and PWD [14, 16]. In addition to the above domains, PWS have more prominent impairment in the domain of social cognition [14], this may be used to differentiate PWS from PWBD and PWD.

Cognitive impairment in people with SMDs is associated with poor functional and clinical outcomes [17–21]. A recent study also showed that cognition worsens gradually if no intervention is provided [22]. Measuring cognition of people with SMDs with robust instruments is important, since measurement and assessment is the first step to intervention. For this purpose, several measures of cognition have been developed and validated in people with SMDs. Although several measures exist, most of these have been developed in Western countries and not always adapted well for use in low-income settings [23–28]. Norms for low- and middle-income countries (LMICs) also do not always exist, making the use and interpretation of these measures complex. In addition, it is not clear which measures would be better candidates for adaptation to low-income setting, since there is no previously synthesized report about the measurement properties of measures adapted in LMICs. Furthermore, most cognitive measures require literacy to respond to the items. Therefore, separate review of validation studies conducted in LMICs can show readers which measure is more appropriate for adaptation in countries with low literacy rate. Finally, multiple languages are spoken in most LMICs as a result, and a separate review of validation studies conducted in LMICs may show readers which measure is adapted across different LMICs speaking different languages.

Although there are numerous studies on the validation of cognitive measures in people with SMDs, only one systematic review has addressed this [29], and there is no previous systematic review focusing on this issue in LMICs. This review is important, because it can help researchers and clinicians to choose the most appropriate measure for their context. As a result, this systematic review is aimed to fill this gap by reviewing the psychometric properties of cognitive measures adapted or developed and validated among people with SMDs in LMICs.

## Methods

We followed the Preferred Reporting Items for Systematic Reviews and Meta-Analyses (PRISMA) guideline to conduct and report this systematic review [30]. We registered the protocol on Prospective Register of Systematic Reviews (PROSPERO) before we started the search (registration number: CRD42019136099).

### Databases searched

PubMed, Embase, PsycINFO, Global Index Medicus, and African Journals Online (AJOL) were searched from the date of inception of the databases until June 07, 2019. Google Scholar was used for forward and backward-searching. We conducted backward-searching on 3rd September 2019 and forward-searching on 29th September 2019.

### Search strategy

We used free terms and controlled vocabulary terms for four keywords: SMDs, cognition, psychometric properties, and LMICs. We combined these four keywords with the Boolean term “AND”. For the complete search strategy, see online resource 1. To increase our chance of capturing all measures validated for the assessment of cognition in people with SMDs, we conducted a forward and backward search. In addition to our registered protocol, we consulted experts in the area by emailing the final list of measures identified for potentially missed measures.

### Eligibility criteria

This review considered studies aimed at developing/adapting and validating a cognitive measure in people with SMDs aged 18 years and older in LMICs. Diagnoses of the disorders needed to be confirmed using either Diagnostic and Statistical Manual of mental disorders (DSM) [31], International Classification of Diseases (ICD) [32], or other recognized diagnostic criteria. For this study, SMDs included schizophrenia, bipolar disorder, and depressive disorders. We chose these three groups of disorders, because cognitive impairment is prominent. We excluded normative studies and adaptation studies involving only healthy participants. Although a normative study is an important step in the adaptation of measures, our aim was to focus on evaluating measures validated in people with SMDs.

We included any measure which was used to assess at least one domain of cognition. Both performance-based (instruments that evaluate behavior on a task or performance) and interview-based (instruments in which the examiner scores the performance through clinical interviews) measures were included.

In this review, a validation study was operationally defined as any study conducted with the aim of evaluating the psychometric properties of a measure, i.e., a study with the main objective of reporting different dimensions of reliability and validity. We also included studies which reported the process of adaptation or development of a measure in people with SMDs without reporting psychometric properties of those measures. Studies only from LMICs were included in this review. We used the World Bank list of economic status of countries during the 2018/2019 financial year as a reference for categorizing countries. Only studies published in English with no restriction in study design were included in the review.

### Full-text identification process

We merged articles found from the databases and removed duplicates. Two of the authors (YG, AD) independently screened each article for eligibility using their title and abstract, followed by full-text screening. Disagreements between the two screeners were resolved by consensus.

### Data extraction

The first author (YG) extracted data from the included articles using data extraction tool developed a priori, and another author (AD) checked all the extracted data for correctness of the extraction. The extraction tool was developed in consultation with the senior authors, referring to previous published systematic reviews, and the requirements for quality assessment followed by piloting it on two articles (the data extraction template is in online resource 2). The core components of the data extraction tool were:

- Authors’ name and affiliation, date of publication, and country
- Study design
- Type of the study (development, adaptation, validation)
- Mode of administration (interview-based vs performance-based)
- Total number of participants in each group (control vs patients)
- Sociodemographic characteristics (age, gender, educational status, and language)
- Duration to administer the tool
- Specific cognitive domains addressed and the number of items of the measure and the domains/sub-tests
- Psychometric properties reported, method of analysis, and findings
- Elements of the quality assessment tool (described in detail on the quality assessment section below)

### Risk of bias/quality assessment

YG and AD independently assessed the risk of bias of individual studies using the COnsensus-based Standards for the selection of health Measurement INstruments (COSMIN) checklist [33]. Any disagreements between the two authors were resolved by discussion. There were no disagreements beyond the consensus agreement between the two screeners. Unlike the registered protocol, we used the updated version of COSMIN checklist, in conducting this review.

COSMIN has a total of 10 boxes for 10 different psychometric properties (each box has 3–35 items). Four dimensions of scoring options are available for each item (i.e., very good, adequate, doubtful, and inadequate). A summary of quality per measurement property is given for each study by taking the worst result for each criterion (for each measurement property addressed) [36]. Since the studies included were not homogenous, we were not able to conduct an assessment of publication bias.

### Criteria used to rank order the measures

In addition to our registered protocol, we evaluated and ranked cognitive measures validated in PWS using five criteria that we developed by adapting from previous reviews [37–41]. Our main reason for the rank ordering the measures is to recommend better measures for adaptation in other settings (it is not for quality assessment). The criteria used to rank the measures were:

1. The number of studies that adapted/validated the measure: one point was given to each measure by counting the number of studies which reported information about the specific measure. According to this criterion, higher score was given to a measure adapted by many studies.
2. Year of publication of studies adapted/validated the measure: in addition to number of studies, year of publication was considered to reduce the risk of recommending a measure which was adapted by many studies, just because it was developed earlier than others. A score of 5 was given for studies published in 2015 and after, while a score of one was given for studies published before 1980. For measures evaluated in more than one studies, the average of the publication year scores was taken.
3. The number of domains the measure addressed: we scored this by counting the number of specific domains that the measure consisted. A single score was given by counting the number of domains that the measure holds from list of domains thought to be impaired in PWS as reported in the systematic review of Nuechterlein et al. [14].
4. Duration to administer: we scored from one to three inversely, i.e., three for brief measures taking 30 min or less, two for measures which take between 30 and 60 min, and one for measures which take more than 60 min to administer.
5. The number of psychometric properties addressed and findings: we added this criterion, since we wanted to consider the number of psychometric properties evaluated for the measure and findings reported. For this criterion, a scale from one to eight was used, where the maximum score was given if five or more measurement properties from COSMIN’s list were evaluated and reported excellent findings and the least score was given if less than two measurement properties were evaluated with less than excellent findings of any of the properties. We have not considered the COSMIN quality rating in this criterion, we only considered the number of measurement properties evaluated from COSMIN’s list and the findings reported. According to this criterion, a better measure is a measure on which many measurement properties have been evaluated and all had been scored excellent findings.

If the necessary information was not contained in the studies included, we gave a score of zero (not reported). The overall ranking of the measures was based on the total sum of scores according to the above five criteria. The highest total possible score is 28 with higher scores indicating a better measure for the recommendation.

### Data synthesis

We used a narrative synthesis to report the findings. For each identified measure, we reported psychometric properties, duration to administer, and other important outcome points that we extracted [e.g., population on which the measure was evaluated, type of the measure (performance- or interview-based), cognitive domains, number of items, etc.]. We also reported the methodological qualities of each study for the specific measurement properties reported.

In addition to our registered protocol, we summarized and synthesized findings. Since the purpose of summarizing was for the aim of general tool selection, we used the updated criteria for good measurement properties in COSMIN systematic review for patient report outcome measurement manual version 1 released in February 2018 [33]. With regards to internal consistency, we graded a Cronbach’s *α* of ≥ 0.7 as excellent, and < 0.7 as satisfactory. For test–retest assessment, intra-class correlation coefficient (ICC) ≥ 0.7 was considered as high, while < 0.7 was considered as poor. For tests with Pearson correlation (*r*) (for test–retest reliability, convergent, or concurrent validity), we used Cohen’s classification and assigned ≥ 0.5 as large, *r* between 0.3 and 0.49 as medium, and between 0.1 and 0.29 as small. Furthermore, we used the COSMIN criteria for summarizing evidence and grade the quality of evidence per measurement properties for measures validated in PWS in more than one study [33].

We reported results for performance-based and interview-based measures separately. We also compared measures validated in PWS with measures validated in PWD and PWBD. It was not possible to conduct meta-analysis and meta-regression because of heterogeneous findings in terms of the measures included and measurement properties reported.

## Results

### Study characteristics

**Fig. 1:**
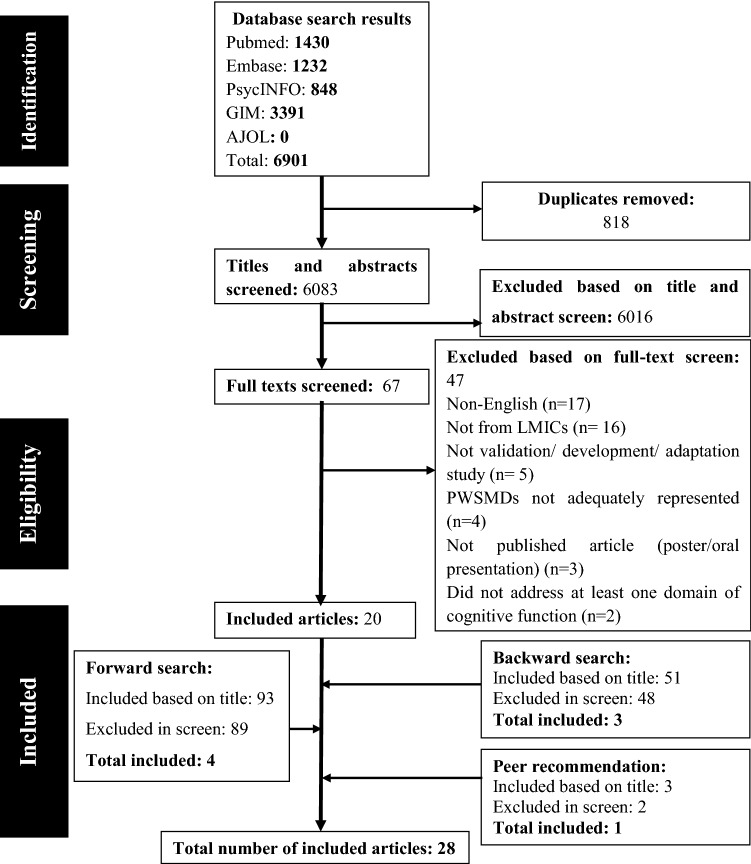
PRISMA (Preferred Reporting Items for Systematic Reviews and Meta-Analysis) flow diagram. *AJOL* African Journals Online, *GIM* Global Index Medicus, *LMICs* Low- and middle-income countries, *PWSMDs* People with severe mental disorders

The search strategy yielded a total of 6091 articles. Title and abstract screening yielded 67 articles. Full-text screening, forward and backward-searching resulted in 27 articles. One article is added later through peer recommendation and the total articles included in this review were 28. Figure 1 shows the flow diagram of article identification. A list of excluded articles with the reason for their exclusion is provided in the online resource 3.

**Fig. 2:**
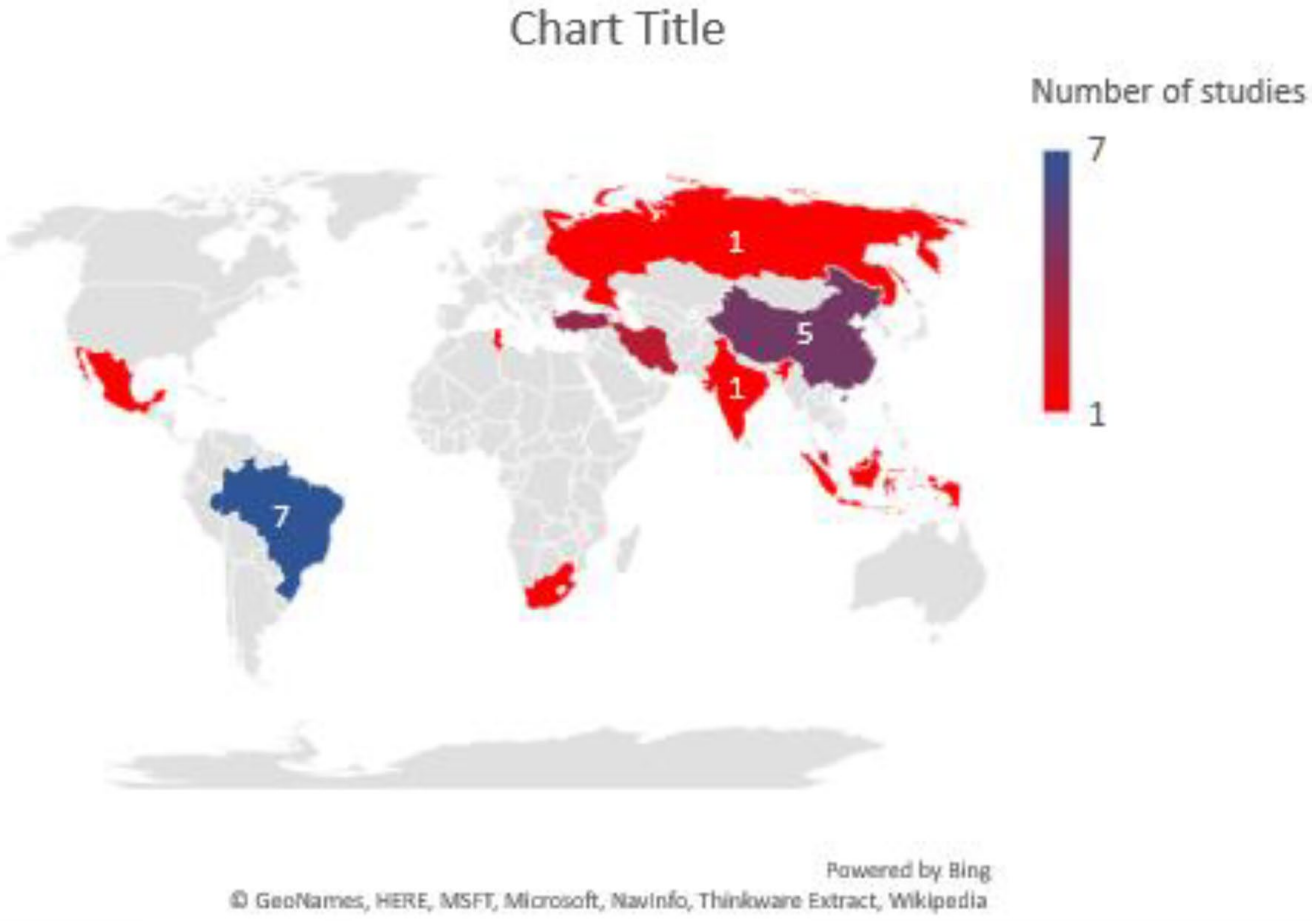
Distribution of included articles in different geographical regions of the world

The 28 studies included in the review evaluated psychometric properties of 23 cognitive measures in people with SMDs from 12 LMICs. Most of these studies were from Brazil (*n* = 7/12) [42–48]. No study was conducted in low-income countries and only three studies were conducted in lower-middle-income countries [49–51] (Fig. 2).

About two-third of the studies (*n* = 18/28) were conducted either in PWS or people with schizophrenia spectrum disorders (PWSSD) with healthy controls (*n* = 13/18) [42, 43, 45, 46, 49, 52–59] or without healthy controls (*n* = 5/18) [47, 50, 51, 59, 60,]. Three studies each were conducted in PWD [44, 62, 63] and PWBD [48, 64, 65] with healthy controls, while those remaining (*n* = 4/28) were conducted in mixed populations [66–69]. A total of 6396 participants (2196 clinical samples and 4200 healthy controls) were included in the main studies of this review. The sample size of PWS/PWSSD in the included studies ranged from 15 to 230 (with a median of 50) for the main studies and 15 to 188 for test–retest reliability. The sample size for healthy controls ranged from 15 to 1757 (with a median of 77) for the main studies and 15 to 84 for test–retest reliability studies. The mean age of PWS/PWSSD participants was 35.2 years. Most studies (*n* = 15/21) had more male participants. On average, PWS/PWSSD had 10.7 years of education. Table 1 describes the participants' characteristics.

**Table 1:** Study characteristics of included articles

Citation (author and year)	Setting (income category) Type of study	Participants (Sample size and type)	Sample characteristics				Name of the measurement
			Age (mean years)	Sex (Male %)	Education (Mean years)	Language	
(Araujo et al. 2015) [42]	Brazil (Upper MI) Validation	PWS = 116; HC = 50 Test retest; PWS = 21; Concurrent PWS = 30	PWS = 38.5; In HC = 39.1 *p* = 0.78	PWS = 51.7%; HC = 46.6% *p* = 0.52	PWS = 8.02; HC = 7.62 *p* = 0.52	Brazilian Portuguese	BACS
(Salgado et al. 2007) [43]	Brazil (Upper MI) Adapt & valid	PWS = 20 HC = 20	PWS = 32.5 ± 8.8 HC = 35.3 ± 12.7 *p* = 0.430	PWS = 50% HC = 50% *p* = 1	PWS = 8.4 ± 3.2 HC = 9.8 ± 2.4 *p* = 0.096	Brazilian Portuguese	BACS
(Mazhari et al. 2014) [56]	Iran (Upper MI) Adaptation & validation	PWS = 50 HC = 50	PWS = 40.5 ± 10.6 HC = 37.3 ± 9.2 *p* = NS	PWS = 60% HC = 50% *p* = NS	PWS = 10.2 ± 2.9 HC = 11.9 ± 3.2 *p* = 0.005	Persian	BACS
(Muliady et al. 2019) [51]	Indonesia (Lower MI) Adapt & valid	PWS = 50	36.48 ± 10.4	84%	14.17 ± 0.3	Indonesian language	BACS
(Abdullah et al. 2013) [60]	Malaysia (Upper MI) Adapt & valid	PWS = 26	28.81 ± 7.8	69.2%	11.27 ± 3.0	Malay	BACS
(Azizian et al. 2011) [52]	Republic of Armenia (Upper MI) Adapt & valid	PWS/PWSZ = 77; HC = 77 Test–retest; PWS = 15 and HC = 15	PWS = 43.6 ± 10.8 HC = 44.2 ± 13.1 *p* = NS	PWS = 52%; In HC = 46% *p* = NS	Mean NR, in PWS = 48% < HS; HC = 44% > HS *p* = NS	Armenian	RBANS
(Dias et al. 2017) [44]	Brazil (Upper MI) validation	LLD = 44; ND = 411	LLD = 81.0 ± 4.8; ND = 80.1 ± 4.7 *p* = 0.142	LLD = 21.2%; ND = 40.6% *p* = 0.009	LLD = 3.9 ± 3.4 ND = 3.8 ± 2.7 *p* = 0.399	Brazilian Portuguese	BCB
(Bosgelmez et al. 2015) [53]	Turkey (Upper MI) Adaptation & validation	PWS/PWSZ = 90 and their caregivers For CI; PWS = 5	PWS = 36.7 ± 9.0 relatives 52.7 ± 13.0	PWS = 75.6%; Relatives 43.4%	PWS = 10.0 ± 3.1; Relatives = 8.1 ± 4.3	Turkish	CAI
(Johnson et al. 2009) [53]	Tunisia (Lower MI) Validation	PWSSD = 105 Pre-test; PWS = 35 + PWSZ = 3 Retest; PWSSD = 39	34 ± 7 Pre-test 34 ± 8.9	81.9% Pre-test 92%	9.7 ± 3.1	Tunisian Arabic dialect	SASCCS
(Mazhari et al. 2017) [55]	Iran (Upper MI) Adapt & valid	PWS = 35; HC = 35	PWS = 30.7 ± 8.3 HC = 30.9 ± 8.4 *p* = NS	PWS = 68.6%; HC = 68.6% = NS	PWS = 12.2 ± 2.8 HC = 12.5 ± 2.6 *p* = NS	Persian	SCoRS
(Aydemir et al. 2017) [62]	Turkey (Upper MI) Adaptation and validation	PWD = 50; HC = 218 (150 HC for FA and; 68 HC for Known group)	PWD = 37.5 ± 11.4 FA group HC 23.4 ± 5.5 *p* < 0.05; Known group HC = 35.4 ± 9.9 *p* > 0.05	PWD = 20% FA group HC = 65.3% Known group HC = 33.8%	Mean NR, PWS = 44.0% PS; FA = 88.7% in university *p* < 0.05; Known group* p* = 35.2% in SS *p* > 0.05	Turkish	PDQ-D and BCCCI
(Shi et al. 2017) [63]	China (Upper MI) validation	129 = PWD; 128 = HC Test–retest = 36 PWD	HC = 34.6 ± 11.8; PWD = 40.6 ± 14.2 *p* < 0.01	HC = 39.8% PWD = 31% *p* = 0.139	HC 14.8 ± 3.5; PWD = 12.8 ± 3.9 *p* < 0.01	NR	PDQ-D
(Ruzita et al. 2009) [57]	Malaysia (Upper MI) Adapt & valid	PWS = 15; HC = 15 Test–retest 30 (PWS = 15 and HC = 15)	38.2 ± 9.5	46.7%	Mean NR, 66.7% in secondary school	Malay	AVLT
(Fonseca et al. 2017) [45]	Brazil (Upper MI) Adaptation & validation	PWS = 99; HC = 99 Test–retest PWS = 45 Pilot study PWS = 15; HC = 15	PWS = 37.6 ± 10.3; HC = 37.6 ± 10.6 *p* = 0.989 Pilot study; PWS = 33.1 ± 8.0; HC = 32.1 ± 7.6; *p* = 0.90	PWS = 52.5%; HC = 52.5% Pilot study; PWS 60%; HC = 60%	PWS = 10.7 ± 3.7; HC = 11.1 ± 3.6 *p* = 0.601 Pilot study; PWS 11.5 ± 3.3; HC = 10.9 ± 3.9 *p* = 0.73	Brazilian Portuguese	MCCB
(Negrão et al. 2016) [46]	Brazil (Upper MI) Adapt & valid	PWS = 44 HC = 152	Total = 26.4; PWS = 39 ± 11; HC = 22 ± 4 *p* < 0.001	Total = 51%; PWS = 59%; HC = 48%; *p* = 0.297	NR	Brazilian Portuguese	SV-FPRT
(Sanvicente-Vieira et al. 2012) [61]	Brazil (Upper MI) Adaptation	For the Pilot 4 PWS was used	NR	50%	all > 7 years of education	Brazilian Portuguese	The ToM Stories and Hinting Task
(Morozova et al. 2017) [42]	Russian Federation (Upper MI) Comparison	PWSSD = 20 (65% PWST; 25% PWSZ; 10% paranoid and subacute psychotic)	26.1 ± 7	65%	Mean NR, 35% = higher education; Minimum = completing SS	NR	Hinting Task', 'Faux Pas', and RMET
(Shi et al. 2019) [58]	China (Upper MI) Development & validation	PWS = 230; HC = 656 Test–retest = 188 PWS	PWS = 38.7 ± 11.5 HC = 39.3 ± 11.4 *p* = 0.525	PWS = 49.5% HC = 50.3% *p* = 0.847	PWS = 10.9 ± 2.9 HC = 10.8 ± 3.2 *p* = 0.828	Mandarin	NBSC
(Zhong et al. 2013) [59]	China (Upper MI) validation	PWS = 60; HC = 58 Test–retest = 33 HC	PWS = 31.47 ± 8.16 HC = 30.83 ± 6.59 *p* = 0.27	PWS = 55.0% HC = 63.8% *p* = 0.33	PWS = 12.4 ± 2.5; HC = 13.9 ± 2.9 *p* = 0.64	Mandarin	CSB
(Changiz et al. 2011) [66]	Iran (Upper MI) validation	PWS + S = 25; PWS -S = 25; PWD = 25, HC = 25	PWS + S = 35.8 ± 5.8; PWS -S = 35.0 ± 6.0; PWD = 32.8 ± 5.3; HC = 33.0 ± 6.1	PWS + S = 72%; PWS -S = 68%; PWD = 64%; HC = 72%	NR	NR, may be Persian	WCST
(Pieters and Sieberhagen, 1986) [68]	South Africa (Upper MI) Validation	PWD = 42; PWMR = 32 and (PWOBS) = 79	No significant difference, but no exact figure	No significant difference, but no exact figure	No significant difference, but no exact figure	NR	SA-WAIS- SF
(Fan et al. 2019) [67]	China (Upper MI) validation	HC = 1757 Clinical (PWS/PWSZ = 119, OCD = 30, PWMID = 90) = 239 Test–retest; HC = 84	HC = 37.8 ± 18.0; OCD = 25.7 ± 5.97 PWS = 35.9 ± 12.1 PWMID = 26.7 ± 9.6; Test retest = 28.1 ± 14.4	HC = 47.5%; OCD = 56.7%; PWS = 47.1%; PWMID = 62.2%; Retest 46.4%	HC = 10.4 ± 3.3; OCD = 13.4 ± 2.6); PWS = 13.0 ± 3.0; PWID = NR	NR, may be Mandarin	SF4- WAIS-IV (FS)
(Gulec et al. 2008) [54]	Turkey (Upper MI) Validation	PWS = 56; HC = 43; The same for Retest	PWS = 29.9 ± 9.3; HC = 27.3 ± 6.3	PWS = 50%; HC = 50%	PWS = 10.1 ± 4.6; HC = 9.6 ± 3.5	Turkish	FAB
(Tuncay et al. 2013) [69]	Turkey (Upper MI) Adaptation and validation	94 d/f cases (33 PWAD, 30 PWIP and 31 PWS); HC = 92 Internal consistency; *n* = 22 Test–retest; *n* = 20	Cases = 62.0 ± 17.3; PWAD = 77.9 ± 4.0; PWIPD 65.3 ± 8.9; PWS = 42 ± 11.5; HC 61.6 ± 14.0 *p* = 0.074	Cases = 50% PWAD = 36.4% PWIPD = 66.7%; PWS = 48.4%; HC = 37%; *p* = 0.877	Mean NR, Cases = 51.1% PS; PWAD = 54.5% PS; PWIPD = 73.3% PS; PWS = 48.4% SS; HC = 46.7% SS; *p* = 0.063	Turkish	FAB
(Xiao et al. 2015) [64]	China (Upper MI) Adaptation & validation	Total = 255 (BD = 125 and HC = 130) Pilot study; BD = 25; Test–retest; BD = 25	BD = 27.3 ± 10.0; HC 28.7 ± 10.7; *p* = 0.45; Test retest = 27.6 ± 9.02	BD = 48.8%; HC = 48.5% *p* = 0.96 Test retest = 56%	BD = 12.4 ± 3.3; HC = 12.1 ± 3.4 *p* = 0.54	NR	COBRA
(Yoldi-Negrete et al. 2018) [65]	Mexico (Upper MI) Validation	HC = 92; PW euthymic BD = 80	HC = 46.8 ± 17.3; BD = 48.1 ± 11.9 *p* = 0.58	HC = 64.1%; BD = 75.0% *p* = 0.12	HC = 18.1 ± 3.5; PWBD = 13.7 ± 3.5 *p* < 0.001	Spanish	COBRA
(Lima et al. 2018) [48]	Brazil (Upper MI) Adaptation & validation	BD = 85 & HC = 65	BD = 49.60 ± 12.9; HC = 45.85 ± 15.7 *p* = 0.121	BD = 28.2%; HC = 22% *p* = 0.240	BD = 10.67 ± 4.0 HC = 14.71 ± 4.1 *p* < 0.001	Brazilian Portuguese	COBRA
(Mehta et al. 2011) [49]	India (Lower MI) Adaptation & validation	Known group (PWS = 9 and HC = 9 for ToM and AB; and PWS = 20 and HC = 20 for SCRT) Concurrent HC = 30	Known group (PWS = 30 (1.2) and HC = 29 (1) for ToM and AB; and PWS = 33.8 (12.8) & HC = 30.9 (9.) for SCRT) Concurrent HC = 30	Known group (PWS = 3 F and HC = 3 F for ToM and AB; and PWS = 7 F and HC = 5 F for SCRT)	Known group (PWS = 9.9 & HC = 10.3 for ToM and AB; & PWS = 9.3 and HC = 9.5 for SCRT)	Hindi and Kannada	SOCRTIS

*AB *Attributional bias, *Adapt & valid* Adaptation and validation, *AVLT* Auditory verbal learning test, *BACS* Brief Assessment of Cognition in Schizophrenia, *BCB* Brief cognitive battery, *BCCI* British Columbia Cognitive Complaints Inventory, *BD* People with bipolar disorder, *CAI* Cognitive Assessment Interview, *CI* Cognitive interview, *COBRA* Cognitive Complaints in Bipolar Disorder Rating Assessment, *CSB* CogState Battery, *FA* Factor Analysis, *FAB* Frontal Assessment Battery, *HC* Healthy Control, *HS* High school, *Lower MI* Lower middle income, *LLD* Late-Life Depression, *MCCB* MATRICS Consensus Cognitive Battery, *MI* middle income, *NBSC* New cognitive battery for patients with schizophrenia in China, *ND* Non-depressed, *NR* Not Reported, *NS* Not significant, *OCD* People with Obsessive–compulsive disorder, *PDQ-D* Perceived Deficit Questionnaire-Depression, *PS* Primary School, *PWAD* People with Alzheimer diseases, *PWD* People with Depression, *PWS* People with schizophrenia, *PWIP* People with Idiopathic parkinsonism, *PWSSD* People with schizophrenia spectrum disorder, *PWMID* People with mild intellectual disability, *PWMR* people with mental retardation, *PWOBS* People with an organic brain syndrome, *PWS + S* People with schizophrenia with positive symptom, *PWS−S* People with schizophrenia with negative symptom, *PWST* People with schizotypal, *PWSZ* People with schizoaffective, *RBANS* Repeatable Battery for the Assessment of Neuropsychological Status, *RMET* Reading the Mind in the Eyes tests, *SASCCS* Self-Assessment Scale of Cognitive Complaints in Schizophrenia, *SA-WAIS-SF* F- South African Wechsler Adult Intelligence Scale Short form, *SCoRS* Schizophrenia Cognition Rating Scale, *SCRT* Social Cue Recognition Test, *SF4-WAIS-FS* the four-subtest index-based short form of Wechsler Adult Intelligence Scale Full scale, *SF4-WAIS-FS* the four-subtest index-based short form of Wechsler Adult Intelligence Scale Full scale, *SOCRTIS* Social Cognition Rating Tools in Indian Setting, *SS* Secondary School, *SV-FPRT* Short version of the Faux Pas Recognition, *ToM* Theory of Mind, *Upper MI* Upper middle income, *WCST* The Wisconsin Card Sorting Test

### Description of the measures

Twenty-three cognitive measures were identified from the 28 studies included. Of these, 15 were evaluated in PWSSD, three in PWD, one in PWBD, and one in PWS and PWD, while three measures were evaluated in a mixed population. The identified measures addressed either single domain of cognition or as many as seven domains, with duration to administer ranging from 10 to 90 min.

Of the measures identified, 17 were performance-based and 12 were evaluated in PWSSD, one each in PWD; and in PWS and PWD, and three in a mixed population. About half of these measures (*n* = 8/17) addressed only neurocognition domains and six addressed social cognition, while three included domains of both neurocognition and social cognition. About two-third of the measures were batteries (*n* = 11/17), while six were single-domain tests.

Six of the 23 measures identified were interview-based. Out of these, three were evaluated in PWSSD, two in PWD, and one in PWBD. Except for one, all these measures addressed neurocognitive domains only (*n* = 5/6). See Table 2 for detailed description of the measures.

**Table 2:** Description of the measures identified from the included articles

Name of the measure	Citation (Author and year))	Type of the measure (Domain)	Duration to administer	Other description (number of items/sub-tests, scoring, and total score) of the measure
BACS	(Araujo et al. 2015) [42] (Salgado et al. 2007) [43] (Mazhari et al. 2014) [56] (Muliady et al. 2019) [51] (Abdullah et al. 2013) [60]	Performance-based (VM, WM, MS, VF, Attention and SP, and RPS)	Average **37.4 min** in **PWS** (40; 43.4; 39.7; 22.1 & 41.7) & (40; 40.5 & 31.5) **37.3 in HC**	Have different tasks under six sub-tests addressing 7 domains of cognition. The test has two alternative versions: version A & B. Translated in about 30 languages. Composite score will be calculated by summing z-score for each test. lower score reflects worse impairment
RBANS	(Azizian et al. 2011) [52]	Performance-based (IM, Visuospatial/Constructional, Language, Attention, DM)	30 min in PWS and 20 min in HC	Has 12 sub-tests, under five scaled indexes. Scores per sub-scale which summed to give a total score. Lower score reflect grater impairment
RMET	(Morozova et al. 2017) [61]	Performance-based (ToM)	NR	Has 37 items/pictures one of this is a trial. Can be scored as one total score or as positive, negative, and neutral emotions. It is a non-verbal test of ToM
Faux Pas test	(Morozova et al. 2017) [61]	Performance-based (ToM)	NR	Is a verbal test of ToM. Has 20 items/situations (10 with irrelevant verbal or non-verbal behavior, and 10 control situations.) Maximum points for the 10 Faux Pas-containing situations is 60 (6/each) whereas 20 points for correctly answered control questions (2 per each)
SV-FPRT	(Negrão et al. 2016) [46]	Performance-based (ToM)	NR	10 stories were selected from the 20 stories of the long version. The selected stories are story 2, 3, 5, 8, 11, 14, 16, 17, 18, and 20
Hinting Task	(Morozova et al. 2017) [61] (Sanvicente-Vieira et al. 2012) [47]	Performance-based (ToM)	NR	The Hinting Task comprised of 10 short sketches or stories The test asked the person to describe the intention of the person presented to them. Each correct response is evaluated as 2 points. The total maximum result is 20 points
The ToM Stories	(Sanvicente-Vieira et al. 2012) [42]	Performance-based (ToM)	NR	It is composed of six sketches or stories
SCoRS	(Mazhari et al. 2017) [55]	Interview-based (Memory, WM, attention, RPS, language and motor skills)	15 min per interview	Has 20 items. Each item scores in a Likert scale ranges from 1 to 4. Rating of not applicable is also possible. A higher score reflects greater impairment A global rating score from 1 to 10 given by the interviewer on the overall level of the impression of the patient’s cognitive difficulty
BCCCI	(Aydemir et al. 2017) [62]	Interview-based (Concentration, memory, trouble expressing thoughts, word-finding, slow thinking, and difficulty PS)	NR	Has 6 items. Scored in a 4-point Likert scale for 0 to 3. Higher score reflecting greater impairment
PDQ-D	(Aydemir et al. 2017) [62] (Shi et al. 2017) [63]	Interview-based (Attention/concentration, retrospective memory, prospective memory, & planning/ organization.)	NR	Has 20 items about the subjective measure of cognitive dysfunction. Scored in a 5-point Likert scale for 0–4. The total score ranges from 0–80. Higher score reflecting impairment
CAI	(Bosgelmez et al. 2015) [53]	Interview-based (VL, WM, RPS, SP, attention/ vigilance, and SC)	Total = 36. 6 min (pt 18.7 & informant 18.0)	Has 10 items. Scored in a 7-point Likert scale from 1 to 7. High scores show poor cognitive status. Patient’s, relative’s and the interviewer’s assessment are scored separately The scale gives the general severity of cognitive impairment scored from 1 to 7
SASCCS	(Johnson et al. 2009) [50]	Interview-based (Memory, attention, EF, language and praxia)	15 min	Has 21 self-rated Likert type questions. The total score is the sum of the individual response. The higher the score the greater the impairment
COBRA	(Lima et al. 2018) [48] (Xiao et al. 2015) [64] (Yoldi-Negrete et al. 2018) [65]	Interview-based (EF, SP, WM, VL and memory, attention/ concentration, and mental tracking)	NR	Has 16 self-reported items, the scale assesses subjective cognitive deficits in bipolar disorder. Each item scored in a 4-point Likert scale from 0 to 3, a total score ranges from 0 to 48 higher score reflecting greater impairment
FAB	(Gulec et al. 2008) [54] (Tuncay et al. 2013) [69]	Performance-based (EF)	10 min	Has 6 sub-tests, each item rated in a Likert scale from 0–3, a total score ranges from 0–18. The higher score showed better performance
AVLT	(Ruzita et al. 2009) [57]	Performance-based (VM)	NR	Has 15 items under five categories
MCCB	(Fonseca et al. 2017) [45]	Performance-based (SP, Attention/concentration, WM, VL and memory, visual learning and memory, RPS, and SC)	90 min	Consisted of 10 tests and 62 sub-tests across seven main domains of cognition thought to be impaired in PWS. Composite score is calculated by summing t-score for each domains
NBSC	(Shi et al. 2019) [58]	Performance-based (attention, SP, VL and memory, visual learning and memory, working memory, RPS, and SC)	NR	This new battery contains 4 measures from MCCB and 5 new measures (Trial making A, BACS, HVLT-R learning and recall, CPT-IP, dominant hand Grooved Pegboard, Color Trails I and II, PASAT)
CSB	(Zhong et al. 2013) [59]	Performance-based (SP, Attention/concentration, WM, VL, and memory, visual learning and memory, RPS, and SC)	40 min	It is a computer-based test, which has 8 tasks that address all seven domains of MATRICS. Composite score is calculated by comparing with the mean and standard deviation of controls
WCST	(Changiz et al. 2011) [66]	Performance-based (Abstract thinking)	NR	Has 128 response cards, and 4 stimulus cards It uses 64 cards in two successive trials
BCB	(Dias et al. 2017) [44]	Performance-based (EF, SP, visuospatial functions, IM, immediate and learning memory, and delayed recall memory)	NR	The test is the combination of category fluency, clock drawing, and figure memory tests
SF4-WAIS-IV	(Fan et al. 2019) [67]	Performance-based (NR, may be information processing, and attention and concentration and may be also EF)	HC = 29.0; POCD = 28.7; PWS = 32.9; and PWMID = 23.1 min	The short form included Block Design (BD), Information (IN), and Arithmetic (AR)
SA-WAIS-SF	(Pieters and Sieberhagen, 1986) [68]	Performance-based (IQ, which is a global cognition)	NR	Is composed of Collidge’s and Golden SF. Coolidge’s shortened form consists of the Digit Symbol, Similarities, Comprehension, and Picture Completion Subtests Golden used the Similarities, Block Design, Digit Symbol, and Object Assembly sub-tests
SOCRATIS	(Mehta et al. 2011) [49]	Performance-based (SC domains (i.e., ToM, social perception and AB))	NR	Is composed of the following tests; i.e. ToM tests including (1) two first order tasks [Sally–Anne & Smarties task] (2) two second order tasks [Ice cream van & Missing cookies story] (3) Metaphor-irony task [Metaphor-Irony stories] & (4) Faux pas task [FPRT]; AB test IPSAQ; & Social perception test [SCRT]

*AB* Attributional bias, *AVLT* Auditory verbal learning test, *BACS* Brief Assessment of Cognition in Schizophrenia, *BCB* Brief cognitive battery, *BCCCI* British Columbia Cognitive Complaints Inventory, *CAI* Cognitive Assessment Interview, *COBRA* Cognitive Complaints in Bipolar Disorder Rating Assessment, *CPT-IP* Continuous Performance Test-identical pairs version, *CSB* CogState Battery, *DM* Delayed Memory, *EF* Executive function, *FAB* Frontal Assessment Battery, *FPRT* The faux pas recognition test, *HVLT-R* Hopkins Verbal Learning Test-Revised, *HC* Healthy Control, *IM* Immediate Memory, *IN* Incidental Memory, *IPSAQ* Internal, Personal, and Situational Attributions Questionnaire, *MCCB* MATRICS Consensus Cognitive Battery, *MS* Motor Speed, *NBSC* New cognitive battery for patients with schizophrenia in China, *NR* Not reported, *PASAT* Paced Auditory Serial Addition Task, *PDQ-D* Perceived Deficit Questionnaire-Depression, *PS* Problem Solving, *PWMID* People with Mild Intellectual Disability, *PWOCD* People with Obsessive Compulsive Disorder, *PWS* People with schizophrenia, *RBANS* Repeatable Battery for the Assessment of Neuropsychological Status, *RMET* Revised Reading the Mind in the Eyes Test, *RPS* Reasoning and Problem Solving, *SASCCS* Self-Assessment Scale of Cognitive Complaints in Schizophrenia, *SA-WAIS-SF* South African Wechsler Adult Intelligence Scale short form, *SC* Social Cognition, *SCoRS* Schizophrenia Cognition Rating Scale, *SCRT* Social Cue Recognition Test, *SF4-WAIS-IV* the four-subtest index-based short form of Weschler Adult Intelligence IV revision, *SOCRATIS* Social Cognition Rating Tools in Indian Setting, *SP* Speed of Processing, *SV-FPRT* Short version of the Faux Pas Recognition Test, *ToM* Theory of Mind, *VF* Verbal Fluency, *VL* Verbal Learning, *VM* Verbal Memory, *WCST* The Wisconsin Test Card Sorting Test, *WM* Working Memory

### Psychometric properties evaluated

These are summarized in Table 3. The most commonly studied psychometric property was hypothesis testing, including convergent, concurrent, and known group validity (evaluated in 25 studies), followed by internal consistency reliability (evaluated in 20 studies), and cross-cultural validity (evaluated in 15 studies). Test–retest reliability was conducted in 14 studies, whereas structural validity was conducted in 11 studies. The least reported measurement property was content validity (*n* = 3/28), followed by criterion validity (*n* = 4/28). None of the included studies evaluated responsiveness to change or measurement error. Very few (*n* = 6/28) studies reported other measurement properties, such as face validity (*n* = 1/28), learning effects (*n* = 1/28), tolerability/feasibility (*n* = 2/28), floor and ceiling effects (*n* = 2/28), comparison of measures (*n* = 1/28), or cross-cultural comparisons (*n* = 1/28).

**Table 3:** Psychometric properties reported in the included articles

Citation (Author and year)	Name of the measure	Reliability	Validity	Other
(Araujo et al. 2015) [42]	BACS	*IC:* Excellent (Cronbach’s *α* = 0.87) *Retest:* High (ICC > 0.7)	*Structural:* One factor *Known group: *Good in differentiating PWS from HC *Concurrent:* Good correlation with standard battery	
(Mazhari et al. 2014) [56]	BACS	*IC:* Excellent (Cronbach’s *α* = 0.74 in PWS and 0.72 in HC)	*Concurrent: *** S**ignificant and moderate to high correlation b/n sub-tests. Good Correlation with the standard battery *Convergent:* No correlation with CPZ equivalent dose (*p* > 0.1) *Known group:* Good ability to differentiate PWS from HC (*p* = 0.01 for all tests) *Structural:* One factor *Cross-cultural:* Persian version of BACS *Criterion:* At cut-off—0.53 has a sensitivity of 0.98 and specificity of 0.66 (ROC = 0.95 CI (0.91–0.99), *p* ≤ 0.01)	
(Salgado et al. 2007) [43]	BACS	*IC:* Excellent (The Cronbach’s* α* = 0.89)	*Known group:* All tests demonstrated significant differences between PWS and HC (*p* < 0.01) Cross-cultural: Brazilian Portuguese version of BACS	**Ceiling and floor effect:** minimal
(Muliady et al. 2019) [51]	BACS	*IC:* Excellent (Cronbach’s* α* = 0.94 for the composite & 0.81–0.91 for sub-tests) *Retest:* High **(**ICC = 0.94 for composite and 0.79–0.93 for sub-tests)	*Concurrent:* very weak until large (0.01 – 0.59) correlation and most are not significant *Cross-cultural:* Indonesian version of BACS	
(Abdullah et al. 2013) [60]	BACS	*Retest:* High (ICC = 0.89 for composite score and 0.76—0.80 for sub-scales) *Inter-rater:* Excellent (*r* = 0.9 – 1.0)	*Cross-cultural:* Malay version of BACS	
(Azizian et al. 2011) [52]	RBANS	*IC:* Excellent (Cronbach’s* α* = 0.92) *Retest:* Low to high (ICC in PWS 0.62 to 0.84; in HC 0.64 to 0.89)	*Convergent:* Correlated with age and education *Known group:* Good ability to differentiate PWS from HC *Cross-cultural:* Armenian version of RBANS	
(Johnson et al. 2009) [50]	SASCCS	*IC:* Excellent (Cronbach’s* α* = 0.85) *Retest:* High (ICC = 0.77)	*Structural:* Six factors *Convergent:* Was not significantly correlated to any of the PANSS score	
(Mazhari et al. 2017) [55]	SCoRS		*Concurrent:* Large and **s**ignificantly associated with the composite score of the BACS (*r* = 0.63 to 0.90). The SCoRS global rating score was correlated at highest with the interviewer rating score (*r* = 0.90), at the lowest with the patient rating score (*r* = 0.40), and the informant rating score was placed in between (*r* = 0.80) *Convergent:* Significantly correlated with GAF and the physical domain of WHO-QoL *Known group:* Good ability to differentiate PWS with HC *Cross-cultural:* Persian version of SCoRS	
(Bosgelmez et al. 2015) [53]	CAI	*IC:* Excellent (Cronbach’s* α* = 0.97 for patient score, 0.91 for relatives, and 0.93 for interviewer)	*Concurrent:* Small to large correlation with the related neurocognitive test (*r* = 0.24–0.56; *p* < 0.05) *Convergent:* Large and **s**tatistically significant correlations with GAF (*r* = -0.538, *p* < 0.001), and social functioning (*r* = -0.520; *p* < 0.01) *Cross-cultural:* Turkish version of CAI-TR	
(Changiz et al. 2011) [66]	WCST		*Known group:* Good ability to differentiate PWS, PWD, and HC *Convergent:* Greater positive or depressive symptoms were not associated with poorer scores on WCST performance. Negative symptom score was the only predictor for the perseverative error scores, (R2 = 0.46, F = 19.57, *p* < 0.001)	
(Dias et al. 2017) [44]	BCB		*Known group:* Good in ability of differentiating b/n PWLLD and PWoutD *Convergent:* Depression severity was negatively correlated with incidental memory (ρ = − 0.412; *p* = 0.003) and positively correlated with FAQ score (*ρ* = 0.308; *p* = 0.035)	
(Aydemir et al. 2017) [62]	BCCCI	*IC:* Excellent (Cronbach’s* α* = 0.93)	*Structural:* One factor *Concurrent:* Moderate and significant correlation with DSST [*r* = 0.40 (*p* < 0.001)] *Known group:* Good ability to differentiate PWD from HC *Cross-cultural:* Turkish version of BCCCI	
	PDQ-D	*IC:* Excellent (Cronbach’s* α* = 0.96)	*Structural:* One factor *Concurrent:* Moderate and significant correlation with DSST [*r* = 0.41 (*p* < 0.001)] *Known group:* Good ability to differentiate PWD from HC *Cross-cultural:* Turkish version of PDQ-D	
(Shi et al. 2017) [63]	PDQ-D	*IC:* Excellent for the total scale and three proposed sub-scales (α = 0.795–0.948) *Retest:* High across total scale and sub-scales (*r* = 0.724–0.865, as well as, ICC = 0.854 to 0.964)	*Structural:* Three-factor structure *Convergent:* Significant correlations with self-reported impaired work productivity, disability in all sub-domains of SDS, and PHQ-9. But, no correlation with DSST *Known group:* Good ability to differentiate PWD from HC	
(Ruzita et al. 2009) [57]	AVLT	*Retest:* low to high (*r* = 0.24–0.84). Good reliability for Trials A5, A1–A5 Total, B1, A6 and A7 (delayed recall), with correlations from 0.69 to 0.84. Lower for Trials A1 (0.23) & recognition (0.24)	*Content:* Good content validity *Structural:* One-factor structure *Known group:* Good ability to differentiate PWS from HC *Cross-cultural:* Malaysian version of AVLT *Face:* Good face validity	
(Pieters and Sieberhagen, 1986) [68]	SA- WAIS- SF		*Concurrent:* High and significant correlations between the FS total IQ and the SF total IQ with all diagnostic groups in both Coolidge (1976) and Golden’s (1976) shortened forms. (*r* = b/n .80 and .91.)	
(Fan et al. 2019) [67]	SF4-WAIS-IV (FS)	*IC:* Excellent (average split-half coefficient is 0.98 for SF4 and 0.94 for the FS) *Retest*: High, 0.91 for FS and 0.90 for SF4	*Concurrent:* Correlated highly with FS (*r* = 0.95) in the standardization sample, and other groups ranged from 0.93 to 0.96 *Convergent:* Females were more likely to be underestimated about 2 scores than males by SF4 when IQs were greater than 111. The closer the group intelligence was to 100, the more accurate the SF4 was in estimating the FS *Criterion:* 93.1% (88.0%) sensitivity (specificity) in HC; 100.0% (84.0) in PWOCD & 94.3% (86.4) in PWS, & 100% classification consistence with IQ level (IQ < 90) in ID The AUC of the stepwise screening in the combined sample was 0.900 (95% CI: 0.885–0.912), with 95.6% sensitivity & 84.3% specificity. PPV was 70.9% & NPV was 98.0%	
(Zhong et al. 2013) [59]	CSB	*IC:* Excellent (Cronbach's* α* = 0.81 in PWS *Retest:* Significant, moderate to large correlation (*r* = 0.39 – 0.62, *p* < 0.05)	*Concurrent:* Large & significant correlation with RBANS in PWS (*r* = 0.54, *p* < 0.001) *Convergent:* Education independently predict CSB composite score *Known group:* Good ability of differentiating PWS from HC (*p* < 0.01) *Structural:* Two factors	
(Fonseca et al. 2017) [45]	MCCB	*IC:* Satisfactory to excellent (for the MSCEIT-ME: Cronbach's α of 0.71; alpha was 0.72 for patients and 0.63 for the control group) *Retest*: Large (r in the 0.70 s & 0.80 s except for LNS & HVLT-R, which were in 0.6 s, & MSCEIT-ME r = 0.55)	*Convergent:*** S**ignificant correlations among all measures that assessed the same construct *Known group:* Good ability to differentiate PWS from HC *Cross-cultural:* Brazilian version of MCCB	**Learning effect:** No **Floor or ceiling effects:** No
(Shi et al. 2019) [58]	NBSC	*Retest*: High (ICC = 0.71–0.94, median = 0.80)	*Known group:* Good ability to differentiate PWS from HC (*p* < 0.01 for all tests except one which is = 0.04 for WCST)	
(Gulec et al. 2008) [54]	FAB	*IC*: Satisfactory (Cronbach's* α* = 0.65) *Retest*: Large (*r* = 0.71, *p* = 0.001)	*Concurrent:* Moderate to large and significant correlation between the CTT first trail, CTT second trail, number of completed categories in the WCST, & preservative errors in the WCST (*r* = 0.58, *p* < 0.001; r = 0.53, *p* < 0.001; r = 0.45, *p* < 0.001; r = 0.77, *p* < 0.001, respectively) *Convergent:* not associated with age & education *Known group:* Good in ability of differentiating b/n PWS and HC	
(Tuncay et al. 2013) [69]	FAB	*IC:* Excellent in total frontal lobe diseases and PWAD (Cronbach’s* α* = 0.73), Satisfactory for PWS & HC (Cronbach’s* α* = 0.66, & 0.52, respectively) *Retest:* Large (*r* = 0.89) *Inter-rater:* Very large (*r* = 1)	*Convergent:* The fewer the years of education, the worse the FAB scale scores (*p* < 0.001); men scored higher than women in a few items Correlation: with standardized Mini Mental Test (0.765, *p* < 0.000) and Stroop scores (may be concurrent) *Known group:* Good ability to differentiate neuropsychiatric subjects and HC *Cross-cultural:* Turkish version of FAB	
(Negrão et al. 2016) [46]	SV-FPRT	*IC:* Excellent (Cronbach’s α in PWS = 0.93, & in HC = 0.94	*Content:* Good content validity *Known group:* Good in ability of differentiating b/n PWS and HC	
(Sanvicente-Vieira et al. 2012) [47]	The ToM Stories & the HT		*Cross-cultural:* Brazilian version of ToM stores and the Hinting task (HT)	
(Morozova et al. 2017) [61]	HT, Faux Pas, & RMET		Comparison of the three tests: RMET is the most difficult, and Hinting task is the least difficult RMET is the most sensitive in detecting ToM. May be used for diagnostic purposes	
(Xiao et al. 2015) [64]	COBRA	*IC:* Excellent (Cronbach's* α* = 0.905) *Retest:* High, (ICC = 0.902)	*Content:* The I-CVIs were b/n 0.83–1.00; average S-CVI (S-CVI/Ave) & universal agreement S-CVI (S-CVI/UA) were 0.97 & 0.81, respectively *Structural:* One factor structure *Concurrent:* No significant correlation with MoCA, except for single measures ((phonemic fluency, *p* = 0.045), VM (delayed recall, *p* = 0.004) *Known group:* Good ability to differentiate PWBD from HC *Cross-cultural:* Chinese version of COBRA *Criterion:* AUC was 0.762. 95% CI: (0.702–0.821). A score of 11 obtains the best balance b/n sensitivity (68.8%) and specificity (81.5%)	*Tolerability:* The percentage of missing values in each of the items contained in the instrument was less than 1%, which showed a high feasibility of COBRA
(Yoldi-Negrete et al. 2018) [65]	COBRA	*IC:* Excellent (Cronbach’s* α* = 0.91)	*Structural:* One-factor structure *Convergent:* Bipolar type II patients had a slightly higher mean COBRA score (15.82 vs 14.64 in type I patients). Although patients were euthymic, HAMD-17 & YMRS scores correlated with COBRA score (Rho = 0.231, *p* = 0.04 & Rho = 0.243, *p* = 0.03). The score is significantly associated in euthymic pt, without antipsychotics and without BzD (Mean COBRA = 11.64 Mann–Whitney U *p* = 0.002; Mean COBRA = 12.81 Mann–Whitney U *p* = 0.046; & Mean COBRA = 13.15 Mann–Whitney U *p* = 0.138; respectively) *Known group:* Good ability to differentiate PWBD from HC	**Cross-cultural comparison** In HC, Mean COBRA score was almost 3 points higher than in the samples from Spain and Denmark In PWBD, mean COBRA score was nearly 2 points below that of Spain and Denmark Adequate congruence coefficients were obtained between the COBRA analyses in the Mexican population and Spanish population (0.96, *p* = 0.01) and acceptable when compared to the Japanese population (0.80, *p* = 0.01)
(Lima et al. 2018) [48]	COBRA	*IC:* Excellent (Cronbach’s* α* = 0.890)	*Structural:* One-factor structure *Concurrent:* No significant correlations were found between the COBRA and objective cognitive measures in the patient group (*p* values > 0.107) In the control group, a negative significant correlation was found between the COBRA and HVLT-R (*p* = 0.006; other *p* values > 0.072) *Convergent* Strong correlation with the cognitive domain of the FAST (*r* = 0.811, *p* < 0.001) Higher COBRA scores were associated with residual depressive (*r* = 0.448; *p* < 0.001) and manic (*r* = 0.376; *p* < 0.001) symptoms, number of depressive episodes (*r* = 0.306; *p* = 0.011), number of total episodes (*r* = 0.256; *p* = 0.038), and suicide attempts (*r* = 0.356; *p* = 0.003) *Known group:* Good ability to differentiate PWBD from HC *Cross-cultural:* Brazilian version of COBRA *Criterion:* AUC was 0.752 indicating good capacity. A score of 10 obtains the best balance b/n sensitivity (64.7%) and specificity (72.3%)	The results showed a high f**easibility/tolerability of the COBRA** since the totality of participants answered all items of the instrument
(Mehta et al. 2011) [49]	SOCRATIS	*IC:* Excellent (Cronbach’s* α* = 0.78)	*Content* Good content validity for SOCRATIS in general. (ToM tasks and AB questionnaire had satisfactory content validity. All the 16 modified vignettes of the SCRT (8 in Hindi and 8 in Kannada) were given a score of > = 4 by > 75% of the experts) *Concurrent* high to low; where ICC were > 0.7 (*p* < 0.001) for social cues in high and low emotion videos and non-social cues in low emotion videos. And it was low (0.36; *p* > 0.05) for the non-social cues in high emotion videos *Known group* Good in ability of differentiating b/n PWS and HC A cut-off value of 0.87 it had 84.2% sensitivity and 81% specify in classifying HC and PWS	

*AB* Attributional bias, *AUC* Area under curve, *AVLT* Auditory Verbal Learning Test, *BACS* Brief Assessment of Cognition in Schizophrenia, *BCB* Brief cognitive battery, *BCCCI* British Columbia Cognitive Complaints Inventory, *BzD* Benzodiazepine, *CAI* Cognitive Assessment Interview, *CI* Confidence Interval, *COBRA* Cognitive complaints in bipolar disorder rating assessment, *CSB* CogState Battery, *CTT* Color trail test, *DSST* Digit Symbol Substitution Test, *EF* Executive function, *FAB* The Frontal Assessment Battery, *FAQ* Pfeffer's Functional Activities, *FAST* Functioning Assessment Short Test, *FAB* The Frontal Assessment Battery, *FS* Full scale, *GAF* Global Assessment of Functioning, *HAMD* Hamilton Depression Rating Scale, *FS* Full scale, *HC* Healthy control, *HT* Hinting task, *HVLT-R* Hopkins Verbal Learning Test revised, *IC* internal consistency, *ICC* Intra Class correlation, *I-CVI* Item-level Content Validity Index, *ID* Intellectual disability, *IQ* Intelligence quotient, *LNS*: Letter-Number Span Test, *MCCB* MATRICS Consensus Cognitive Battery, *MoCA* Montreal Cognitive Assessment Scale, *MSCEIT-ME* Mayer-Salovey-Caruso Emotional Intelligence Test-Managing Emotion, *NBSC* New cognitive battery for patients with schizophrenia in China, *NPV* Negative predictive value, *PDQ-D* Perceived Deficit Questionnaire-Depression, *PHQ-9* Patient Health Questionnaire, *PNSS* Positive and negative syndrome scale, *PPV* Positive predictive value, *PWBD* People with bipolar disorder, *PPV* Positive predictive value, *PWD* People with depression, *PWLLD* People with Late-life depression, *PWoutD* People without depression, *PWOCD* People with obsessive–compulsive disorder, *PWS* People with schizophrenia, *Retest* test–retest reliability, *RBANS* Repeatable Battery for the Assessment of Neuropsychological Status, *RMET* Reading the mind in the eye test, *ROC* Receiver operating curve, *SA* South Africa, *SASCCS* Self-Assessment Scale of Cognitive Complaints in Schizophrenia, *SCoRS* Schizophrenia Cognition Rating Scale, *SCRT* Social Cue Recognition Test, *S-CVI* Scale Level Content validity Index, *SDS* Sheehan Disability Scale, *SF* Short form, *SF4-WAIS-IV* the four-subtest index-based short form of WAIS-IV, *SOCRATIS* Social Cognition Rating Tools in Indian Setting, *SV-FPRT* Short Version of the Faux Pas test, *ToM* Theory of Mind, *VM* Verbal Memory, *WAIS* Weschler Adult Intelligence scale, *WCST* The Wisconsin Test Card Sorting Test, *WHO-QOL* World Health Organization Quality of Life, *YMRS* Young Mania Rating Scale

It was not possible to pool the findings of the psychometric properties reported because of heterogeneity in the measures used, psychometric properties reported, and populations studied. We therefore summarized them in Table 3 and provided a narrative synthesis.

Almost all of the studies reported excellent internal consistency (*n* = 18/20), and most studies reported high test–retest reliability (*n* = 11/14). Only one-third of the studies reported good concurrent validity (*n* = 4/14), whereas good convergent validity was reported by most studies (*n* = 11/15). All of the studies (*n* = 21) which evaluated known group validity reported good ability to discriminate different clinical samples and healthy controls. Likewise, appropriate content validity (*n* = 4), excellent criterion validity (*n* = 4), high tolerability/feasibility (*n* = 2), good face validity (*n* = 1), minimal floor and ceiling effect (*n* = 2), and no learning effect (*n* = 1) were also reported.

Of the studies which evaluated performance-based measures, the majority reported excellent internal consistency (*n* = 8/12), high test–retest reliability (8/11), and good concurrent validity (*n* = 4/8). Good convergent validity was reported in two-third of the studies (*n* = 6/9). Three studies assessed structural validity and two of them reported one-factor structure and the other one reported a two-factor structure. Two studies evaluated criterion validity and reported excellent sensitivity and specificity. Nine different studies evaluated cross-cultural validly and yielded nine different versions of the measures.

Of the studies which evaluated interview-based measures, all reported excellent internal consistency (*n* = 8/8), high test–retest reliability (3/3), and good convergent validity (*n* = 5/6). None of the included studies reported good concurrent validly, whereas two-thirds of the studies reported moderate concurrent validity (*n* = 4/6). Seven studies assessed structural validity and five of them reported one factor, one study reported three factors, and another one reported six factors. Two studies evaluated criterion validity and reported excellent sensitivity and specificity. Six different studies evaluated cross-cultural validly and yielded six different versions of the measures. Two studies evaluated feasibility/tolerability of measures and found that both measures were feasible/tolerable for the respondents (less than 1% of missing values were reported). See Table 3 for details.

#### Summary of evidence per measure

Three studies [47, 61, 62] reported more than one measure, while six measures were described by more than one study. In Table 4, we summarized the psychometric properties of measures reported in more than one study in PWS. For detailed psychometric properties of measures reported in other population and in PWS in only one study, please see Table 3.

**Table 4:** Best evidence synthesis for measures evaluated in more than one study in people with schizophrenia using the COSMIN systematic review for PROM manual version 1 released in Feb 2018

Name of the measure	Findings and evidence	Measurement properties under the COSMIN list							Other (not in COSMIN list)
		Content validity	Structural validity	Internal consistency	Test–retest reliability	Hypotheses testing	Cross-cultural validity	Criterion validity	
BACS	Summarized findings (pooled) *n* = 5 studies [42, 43, 51, 56, 60]	+^ 1^	? (one-factor structure) *N* = 274 *n* = 2 studies	+ (Cronbach’s α of 0.74 to 0.94) *N* = 364 *n* = 4 studies	+ (ICC of 0.7 to 0.94) *N* = 97 *n* = 3 studies	Mixed with most + (Good concurrent, convergent and known group validity) *N* = 364 *n* = 4 studies	? (Persian, Brazilian, Indonesian, and Malay version) *n* = 4 studies	+ (At cut-off − 0.53 has a sen of 0.98 and spec of 0.66 (AROC = 0.95; CI (0.91–0.99) *N* = 100 *n* = 1 study	Ceiling and floor effect Minimal ceiling and floor effect *n* = 1 study
	Quality of evidence		Moderate	High	Low	High	Moderate	Low	
FAB	Summarized findings (pooled) *n* = 2 studies [54, 69]	+^1^		- (Cronbach’s α of 0.52 to 0.65) *N* = 122 *n* = 2 studies	? (r of 0.71 to 0.89) *N* = 119 *n* = 2 studies	+ (Good concurrent, convergent and known group validity) *N* = 285 *n* = 2 studies	? (Turkish version) *n* = 1 study		Inter-rater reliability Very high (*r* = 1)
	Quality of evidence			High	Moderate	High	Low		
Hinting Task	Summarized findings (pooled) *n* = 2 studies [4, 61]	+^1^					? (Brazilian version) *n* = 1 study		Comparison with other measures Least difficult measure compared to RMET and the Faux past test
	Quality of evidence						Low		

*BACS* Brief Assessment of Cognition in Schizophrenia, *COSMIN* COnsensus-based Standards for the selection of health Measurement Instruments, *FAB* Frontal Assessment battery, *RMET* Reading the Mind in the Eye Test

Rating: “ + ” = positive rating, “?” = indeterminate rating,” – “ = negative rating

1. One study examines content validity during cross-cultural validation, but no results were reported (since content validity is necessary, we examined the appropriateness of the items and the evaluation is based on our view of the items’ content)

*Brief Assessment of Cognition in Schizophrenia (BACS):* this performance-based measure was evaluated in five studies [42, 43, 51, 56, 60]. BACS has six sub-tests which address seven domains of cognition, and on average, it takes 37.4 min to administer it in PWS (Table 2).

High-quality evidence was reported for internal consistency (with positive rating from four studies), and hypothesis testing (with mixed result with most reporting good concurrent, convergent, and known group validity from four studies). While moderate-quality evidence was reported for structural validity (with one-factor structure from two studies) and cross-cultural validity (yielded Persian, Brazilian, Indonesian, and Malay version). However, low-quality evidence was reported for test–retest reliability (with positive rating from three studies) and criterion validity (with positive rating from one study). Other than the COSMIN list of measurement properties, minimal ceiling and floor effect were reported in one study (Table 4).

*Frontal assessment battery (FAB):* Two studies [54, 69] evaluated this performance-based measure. FAB has six sub-tests each to be scored in a Likert scale from 0 to 3 with a total score of 0 to 18. A higher score reflects better performance. It takes only 10 min for administration, but it assesses only one domain (i.e., executive function) (Table 2).

High-quality evidence were reported for internal consistency (with negative rating from two studies), and hypothesis testing (with positive rating for good concurrent, convergent, and known group validity from two studies). Whereas moderate-quality evidence was reported for test–retest reliability (with intermediate rating from two studies). However, low-quality evidence was reported for cross-cultural validity [54]. Other than the COSMIN list of measurement properties, very high inter-rater reliability was reported in one study (Table 4).

*Hinting task:* Two studies [48, 61] addressed this performance-based measure of theory of mind. The Hinting task comprised 10 short sketches/stories which focused on assessing the person’s ability to describe the intention of the person from the stories presented (Table 2).

Cross-cultural validity (*n* = 1/2) and comparison with other tests (*n* = 1/2) were evaluated for this measure. The cross-cultural validity of the Hinting task resulted in the Brazilian version which was rated as low-quality evidence, while it was found to be the least difficult measure in detecting theory of mind compared with Reading the Mind in the Eye Tests (RMET) and Faux pas test (Table 4).

### Clinical and research usefulness evaluation

We ranked measures evaluated in PWS using five criteria described in detail in the section “Criteria used to rank order the measures”. Summing the scores for each criterion, BACS ranked first, Cognitive Assessment Interview (CAI) ranked second, and MATRICS Consensus Cognitive Battery (MCCB) and CogState Schizophrenia Battery (CSB) ranked third. When looked at performance-based and interview-based measures separately, BACS, MCCB, and CSB stood out as the top three performance-based measures. While, CAI ranked first from interview-based measures, followed by Schizophrenia Cognition Rating Scale (SCoRS), and Self-Assessment Scale of Cognitive Complaints in Schizophrenia (SASCCS). See Table 5.

**Table 5:** Ranking of the measures evaluated only in people with schizophrenia in the included studies

Name of the measure	Number of studies reporting the measure	Year of publication	Number of domains the measure held	Duration to administer of the measure	Number and evaluation of psychometric properties reported about the measure	Sum	Rank
Performance-based							
BACS	5	4.2	5	2	7	23.2	1
MCCB	1	5	7	1	5	19	3
CSB	1	4	7	2	5	19	3
NBSC	1	5	7	NR	3	16	5
RBANS	1	4	2	3	5	15	7
AVLT	1	3	1	NR	7	12	9
Hinting task	2	4.5	1	NR	4	11.5	10
SV-FPRT	1	5	1	NR	4	11	11
RMET	1	5	1	NR	2	9	12
SOCRATIS	1	4	1	NR	3	9	12
The ToM Stories	1	4	1	NR	2	8	14
Faux Pas test	1	5	1	NR	1	8	14
Interview-based							
CAI	1	5	6	3	6	21	2
SCoRS	1	5	3	3	4	16	5
SASCCS	1	3	3	3	5	15	7

*AVLT* Auditory verbal learning test, *BACS* Brief Assessment of Cognition in Schizophrenia, *CAI* Cognitive Assessment Interview, *CSB* CogState Battery, *MCCB* MATRICS Consensus Cognitive Battery, *NBSC* New cognitive battery for patients with schizophrenia in China, *RBANS* Repeatable Battery for the Assessment of Neuropsychological Status, *RMET* Revised Reading the Mind in the Eyes Test, *SASCCS* Self-Assessment Scale of Cognitive Complaints in Schizophrenia, *SCoRS* Schizophrenia Cognition Rating Scale, *SOCRATIS* Social Cognition Rating Tools in Indian Setting, *SV-FPRT* Short version of the Faux Pas Recognition Test, *ToM* Theory of Mind

Key:

1. Number of studies reporting the measure: a single score was given to the study that addresses the measure

2. Year of publication: 5 = after 2015; 4 = [2010, 2015); 3 = [2000, 2010); 2 = [1980, 2000); 1 = before 1980; for measures with more than one study the average score of year of publications was taken

3. Number of domains the measure held: a single score was given by counting the number of domains that measure holds from list of domains thought to be impaired in people with schizophrenia as reported in the systematic review of (Nuechterlein et al. 2004) [14]

4. Duration to administer: 1 > 1 h, 2: 30–60 min; 3: < 30 min; NR; not reported

5. Psychometric properties: 8** = **five and more measurement properties evaluated with all excellent report; 7 = five and more measurement properties evaluated with less than excellent report; 6 = three or four measurement properties evaluated with all excellent report; 5 = three to four measurement properties evaluated with less than excellent report; 4 = two measurement properties evaluated with excellent report; 3 = two measurement properties evaluated with less than excellent report; 2 = less than two measurement properties evaluated with excellent report; 1 = less than two measurement properties evaluated with less than excellent report

### Methodological quality

**Fig. 3:**
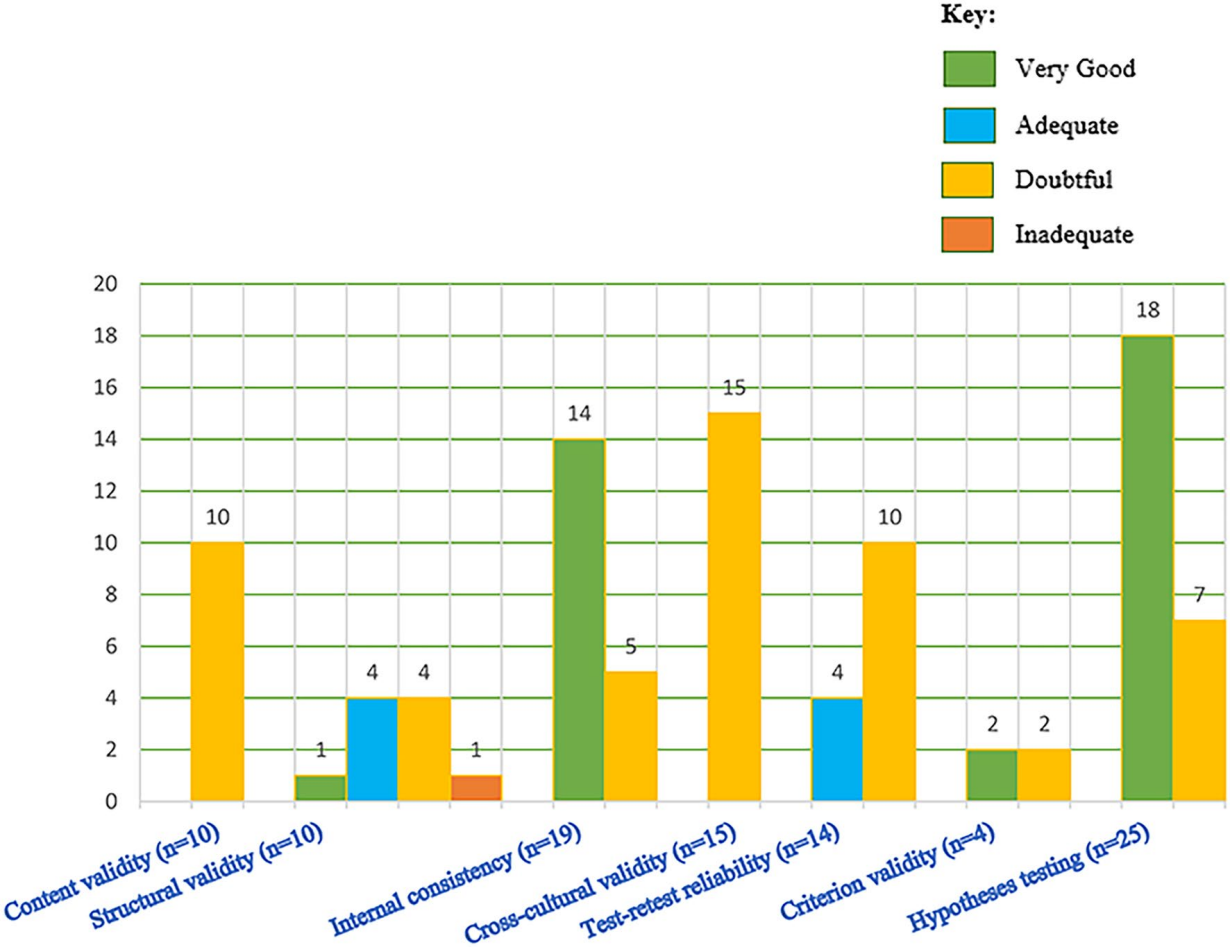
Number of studies with very good, adequate, doubtful, or inadequate-quality rating per each measurement properties addressed

From the ten domains of the COSMIN checklist, two domains (responsiveness to change and measurement error) were not reported in any of the included articles. We have not evaluated patient-reported outcome development check box (box 1), since this study focuses on adaptation studies rather than development studies and most measures included here are not freely available. Most of the included studies reported hypothesis testing (*n* = 25/28). For a summary of the methodological qualities of the included studies, see Fig. 3.

Quality of the included studies ranged from very good to inadequate. Thirteen studies were rated very good quality for internal consistency; while 5/19 studies were rated as doubtful, and one inadequate-quality rating. The main reason for the doubtful rating in those studies was that there were minor methodological problems and the reason for the inadequate-quality rating was that internal consistency was not calculated for each dimensions of the scale. None of the studies that assessed test–retest reliability had very good rating and the reason for this was that it is doubtful that the time interval used was appropriate (5/10 doubtful rating). All the 10 studies that evaluated content validity were found to have doubtful quality. The reason is that in all studies, it is not clear the methodology they followed (not clear whether the group moderator was trained, topic guide was used, group meetings were recorded, etc.). From the 10 studies that evaluated structural validity, one study had very good quality, four studies had adequate quality, another four doubtful rating, and one inadequate quality. The reason for the inadequate rating was that they used a very small sample. Most of the included studies (*n* = 25) examined one form of hypothesis testing (i.e., concurrent, convergent, or known group validity). Most of the studies were rated as very good (*n* = 18/25) and the remaining were rated as doubtful (*n* = 7/25). The main reason for the doubtful rating was that minor methodological problems in sample selection. The quality of all studies that evaluated cross-cultural validity was rated as doubtful (*n* = 15/15). The main reason for this was that multiple group factor analysis was not performed, it is not clear whether the samples are similar, and the approaches used to analyze the data are not clear. Finally, four studies evaluated criterion validity—two very good quality and two doubtful quality. Figure 3 presents the methodological quality scores for each of the included studies. Detailed report of the quality rating for each study at each measurement property is given as online resources 4.

## Discussion

There is limited availability of valid and reliable cognitive assessment and screening instruments for people with SMDs in LMICs. This is partly due to the limited adaptation and validation efforts in the literature. A first step to improving this situation is to systematically assess the current status of the literature and identify cognitive measures which are already validated and may be used and adapted for the assessment of cognition in people with SMDs in LMICs.

This review identified 28 studies and 23 independent cognitive measures. Most of the measures evaluated cognition in PWS from upper-middle-income countries such as Brazil and China. None of the studies was from low-income settings, suggesting that we have no evidence about the psychometric properties of these measures in low-income countries. We found participants’ education level to be high (on average 11 years of education). The majority of the studies had low methodological quality, and based on limited sample size. This was more so in cross-cultural validation and content validity studies.

According to the criteria that we considered for clinical and research usefulness, we recommend BACS, CAI, MCCB, and CSB as most suitable measures to be adapted for the cognitive assessment of PWS in LMICs. Of these, the BACS is the most frequently evaluated measure and it is the measure with most adaptations and reliable psychometric properties. In addition, it addresses comprehensive domains of neurocognition with shorter administering time. Since LMICs have different contexts in terms of cultural, linguistic, economic, and educational backgrounds, we recommend validation studies at different sites with the above three measures as a starting point for validation and adaptation of other measures. Studies focusing on norming of measures in LMICs would also be useful. The use and adaptation of a measure to a new context requires considering carefully how the new version of the measure will interact with the new context and population [70–72]. Assessment of psychometric properties is the result of an interaction between the measure, the context, and the population. Adapting measures to LMICs requires a fundamental shift in each of these aspects, and therefore, the literature and evidence on existing measures can only have a limited value to inform the adaptation process [73, 74].

More than half of the measures included in this review do not assess social cognition (*n* = 13/22) which is a domain usually found impaired in PWS. We recommend clinicians and researchers in LMICs to consider measures that can include this domain, although the different social and contextual factors may be a challenge in developing comparable social cognition tests.

We did not find studies conducted in low-income countries, the majority of the studies were from upper-middle-income countries (e.g., from Brazil and China). This is an important finding as it highlights a clear gap in the literature and availability of cognitive measures globally. The context in low-income countries is different from upper-middle-income and higher-income countries. For example, according to the World Bank, 67% of the total population in lower-income countries are rural residents, who may not be literate [75]. Overall only 63% have basic literacy skills (able to read and write a simple sentence) and may not be familiar with settings outside their local community. LMICs are diverse in terms of educational status, culture, and language. With this in mind, it is important that local experts lead the adaptation efforts on cognitive measures in LMICs, using the recommended measures in this review as an initial point.

The average educational level of PWSSD in the included studies was approximately 11 years, which shows that the findings reported here may not translate directly to low-income settings where the overall literacy rate tends to be lower. Researchers need to consider the effect of education [76–78], culture [79, 80], and language [81] when deciding which measure to adapt and use in LMICs’ context.

It should be borne in mind that a low score on a cognitive test may not always reflect cognitive impairment, but simply lack of familiarity with the material presented. LMICs are also diverse in cultural practice, which should be considered during the adaptation process. For example, one item of SCoRS [82] requires the participant to assess how difficult it is for them to follow a television show. Answering this item has clear economic and cultural implications. Adaptation of this item may require a fundamental rethink in relation to the setting. The other factor that should be considered is language. Again, LMICs are less homogeneous in langue knowledge and use. In many countries in Africa and Asia, multiple languages are spoken within a given country, and it may not be simple to define people’s first language. Using a cognitive measure adapted in a different linguistic context may not be appropriate and non-verbal cognitive measures may be preferred. This further emphasized the question of how much context influence cognitive assessment [83]. The literature shows that different results in a cognitive test can be due to variation in cultural interpretations, such as when a test has items or tasks that are only familiar in certain contexts [79].

This review has a number of strengths in that we included any measure of cognition in people with SMDs with no restriction in the domain of cognition evaluated. We also followed a rigorous protocol, which we preregistered in PROSPERO (https://www.crd.york.ac.uk/PROSPERO/), searched four comprehensive databases without restriction on the date of publication, and used a comprehensive quality assessment tool (the COSMIN criteria) [33].

However, our review has limitations. First, the broad scope of the review makes the data inappropriate for meta-analysis. Our study protocol allowed a wide variety of study outcomes to obtain a broad overview of the field given the paucity of knowledge and lack of prior systematic reviews. Second, the criteria we used to rank the measures were not used previously (even though we adapted them from previous reviews). Third, this review excluded non-English studies, which might limit the generalizability of the findings. Fourth, gray literature was not searched; however, we conducted forward and backward-searching which extended our included studies from 21 to 28. Readers are recommended to consider the generalizability of our review considering those limitations.

Reviewing and systematically assessing the psychometric properties of measures in this field are useful for researchers, clinicians, and policymakers in LMICs and beyond. Since LIMICs are diverse in language, culture, and education, our recommendations may not work for every country, and hence, these need to be contextualized. This review could help researchers in measure selection when planning studies, particularly for adaptation studies. Other potential use includes guiding choices of the best measures in conducting longitudinal studies to assess change in cognition and clinical trials for interventions aiming to improve cognition. In this review, we considered measurement properties such as test–retest reliability, learning effect, tolerability, and practicality which are important in repeated assessment. Therefore, researchers can compare measures on those criteria when looking for the best measures for longitudinal studies and clinical trials. Clinicians in LMICs could use this review to compare different measures and use the one that most suits their specific needs and context. Policymakers can use the results of this study to design prevention and treatment strategies regarding cognition in people with SMDs—such as developing a guideline, integrating routine assessment of cognition in clinical settings, and promoting research activities in the treatment of cognitive impairment. This review points clearly to a gap in the evidence for cognitive assessment for SMDs in LMICs. This may suggest a gap in the use of cognitive assessment in clinical practice and the need for adaptation and validation study to make these tools available to services, clinicians, and service users.

## Supplementary Information

Below is the link to the electronic supplementary material.

Supplementary file1 (DOCX 21 KB)

Supplementary file2 (XLSX 13 KB)

Supplementary file3 (DOCX 124 KB)

Supplementary file4 (DOCX 75 KB)

## Data Availability

All the data used made available in the manuscript and supplementary materials.

## Abbreviations

AJOL: African Journals Online
BACS: Brief Assessment of Cognition in Schizophrenia
CAI: Cognitive Assessment Interview
COBRA: Cognitive complaints in Bipolar Disorder Rating Assessment
COSMIN: COnsensus-based Standards for the selection of health Measurement Instruments
CSB: CogState Battery
DALYs: Disability-Adjusted Life Years
DSM: Diagnostic and Statistical Manual of mental disorders
FAB: Frontal Assessment Battery
ICD: International Classification of Diseases
ICC: Intraclass Correlation Coefficient
LMICs: Low- and Middle-Income Countries
MCCB: MATRICS Consensus Cognitive Battery
NBSC: The New Cognitive Battery for patients with Schizophrenia in China
PDQ-D: Perceived Deficit Questionnaire-Depression
PRISMA: Preferred Reporting Items for Systematic Reviews and Meta-Analyses
PROSPERO: Prospective Register of Systematic Reviews
PWBD: People with bipolar disorder
PWD: People with Depression
PWS: People with Schizophrenia
PWSSD: People with Schizophrenia Spectrum Disorders
RBANS: Repeatable Battery for the Assessment of Neuropsychological Status
RMET: Reading the Mind in the Eye Test
SASCCS: Self-assessment scale of cognitive complaints in schizophrenia
SCoRS: Schizophrenia Cognition Rating Scale
SMDs: Severe Mental Disorders
YLD: Years Lived with Disability
