# Supplementary material for: Assessing cognition in people with severe mental disorders in low- and middle-income countries: a systematic review of assessment measures
Source: Soc Psychiatry Psychiatr Epidemiol. 2021 Jun 18;57(3):435–60. doi: 10.1007/s00127-021-02120-x (PMC8934327; doi:10.1007/s00127-021-02120-x)
Supplement: Supplementary file 1 — Supplementary file1 (DOCX 21 KB) [file 127_2021_2120_MOESM1_ESM.docx]

**Search strategy**

Online resource 1: Search strategy for a systematic review of the psychometric properties of cognitive measures validated among people with severe mental disorders in low and middle-income countries.

| Big terms |  | |
| --- | --- | --- |
| **Big term 1:**  Cognition | Free terms | Cognit* OR neurocogn* OR neuropsych* OR  Attention OR Vigilance OR Memory OR “Working Memory” OR “Verbal Learning” OR “Visual Learning” OR “Verbal Memory” OR “Visual Memory” OR “Speed of Processing” OR “Problem Solving” OR Reasoning OR “Executive function” OR “Processing Speed” OR “Psychomotor Speed” OR “Perceptual Speed” OR “Social Cognition” OR “Theory of mind” OR “Emotion Perception” OR “Social Perception” OR “Attributional Bias” OR “Attributional Style” OR “Emotion Processing” OR “Motor Disorder” OR Vocabulary OR Comprehension OR “Verbal Comprehension” OR concentration OR  MCCB OR “MATRICS Consensus Cognitive Battery” OR SCoRS OR “Schizophrenia Cognition Rating Scale” OR CAI OR “Cognitive Assessment Interview” OR CGI-CogS OR “Clinical Global Impression of cognition in schizophrenia” OR “Brief Assessment of cognition in Schizophrenia” OR BACS OR MSCEIT OR “Mayer-Salovey-Caruso Emotional Intelligence Tests” OR CSSS OR “Cognitive Screening Scale for Schizophrenia” OR SCOPE OR “The Social Cognition Psychometric Evaluation” OR “GEOPTE” OR “Spanish Group for the Optimization and Treatment of Schizophrenia” OR PRECIS OR “Patient Reported Experience of Cognitive Impairment in Schizophrenia” OR ANT OR “Attention Network Test” OR ACL OR “Allen Cognitive Levels” OR “Clock Drawing” OR LUT OR “Letter Updating Test” OR WUT OR “Word Updating Test” OR “Dot test” OR “Wisconsin Card Sorting Test” OR Wisconsin* OR WCST OR Stroop OR “Stroop Color Word Test” OR “Verbal Fluency” OR “Tower of London” OR “Trail Making” OR “Digit Span” OR “Cognitive Estimates Test” OR “Boston Naming Test” OR Categorization OR Wechsler OR, “Wechsler Memory Scale” OR WMS OR “Complex Figure” OR “Face Recognition” OR “Spatial Perception” OR “Line Orientation” OR “Finger Tapping” OR “Continuous Performance” OR CPT OR “Back ward masking” OR IQ OR “Block Design” OR “Word Fluency” OR “affect recognition” OR “Dichotic Listening” OR “Verbal Fluency” OR “California Verbal Fluency Test” OR CVLT OR Token |
|  | Mesh terms | "Cognitive Neuroscience"[Mesh] OR "Neuropsychology"[Mesh] OR "Neuropsychiatry"[Mesh] OR "Neurocognitive Disorders"[Mesh] |
|  | Emtree | exp cognition/ OR exp cognitive defect OR exp neuropsychology/ OR exp neuropsychiatry/ |
|  | PsycINFO terms | exp COGNITION/ OR exp Cognitive Ability/ OR exp NEUROPSYCHOLOGY/ OR exp Cognitive Impairment/ OR exp NEUROPSYCHIATRY/ OR exp NEUROCOGNITION// |
| **Big term 2:**  Schizophrenia | Free terms | Schizop* OR Psychosis OR Psychotic OR “Severe mental disorder” OR “Severe mental illness” OR ScZ OR SMD OR “Positive symptoms” OR “Negative symptoms” OR “Disorganized symptoms” OR Paran* OR Catato* OR “residual symptoms” OR Bipolar OR Manic-depress* OR Mania OR Manic OR cyclotomic OR “Rapid cycling” OR “Affective Psychosis” OR depress* OR “Melancholic Depression” OR “Atypical depression” OR “seasonal depression” OR “Major depressive disorder” OR MDD OR “Major Depression” |
|  | Mesh terms | "Schizophrenia Spectrum and Other Psychotic Disorders"[Mesh] OR "Bipolar and Related Disorders"[Mesh] OR "Depression"[Mesh] OR "Mood Disorders"[Mesh] |
|  | Emtree | exp schizophrenia/ OR exp schizophrenia spectrum disorder/ OR exp "Schedule for Affective Disorders and Schizophrenia"/ OR exp schizoaffective psychosis/ OR exp psychosis/ OR exp bipolar mania/ OR exp mania/ OR exp “bipolar disorder”/ OR exp depression/ |
|  | PsycINFO | exp PSYCHOSIS/ / OR exp Affective Disorders/ OR exp SCHIZOPHRENIA/ OR exp Bipolar Disorder/ OR exp MAJOR DEPRESSION/ |
| **Big term 3:**  Psychometric property | Free terms | “Psychometric property” OR Psychometr* OR Valid* OR Reliabil* OR “Internal Consistency” OR “Inter-rater Reliability” OR “Test-retest reliability” OR “Practice effect” OR “Convergent validity” OR “Concurrent validity” OR “Structural validity” OR “Item difficulty” OR “Item discrimination” OR “Differentiation Item functioning” OR DIF OR “Floor Effect” OR “Ceiling effect” OR “Divergent validity” OR “Discriminant validity” OR “Criterion Validity” OR Sensitivity OR Specificity OR “Construct validity” OR “known group difference” OR “Item response theory” OR IRT OR “factor structure” OR Adaptation OR Development OR Creation OR Design OR acceptability OR feasibility OR Measurement OR Assessment OR Tool OR instrument OR Test OR Performance OR Examination OR Interview OR Scale OR Questio* OR batter* |
|  | Mesh terms | "Psychometrics"[Mesh] OR "Reproducibility of Results"[Mesh] OR "Evaluation Studies as Topic"[Mesh] OR "Evaluation Studies" [Publication Type] OR "Validation Studies" [Publication Type] OR "Consensus Development Conferences as Topic"[Mesh] OR Consensus Development Conference [Publication Type] |
|  | Emtree terms | exp validation process/ OR exp psychometry/ OR exp validity OR exp reliability/ OR exp adaptation/ OR exp instrument validation/ |
|  | PsycINFO terms | exp Measurement/ OR exp Factor Structure/ OR exp Test Validity/ OR exp Test Construction/  OR exp Test Reliability/ OR exp Statistical Reliability/ OR exp Statistical Validity/ |
| **Big term 4**  **LMIC** | Free terms | Developing countr* OR less developed countr* OR third world nation* OR third world countr* OR under developed nation* OR underdeveloped nation* OR under developed countr* OR underdeveloped nation* OR middle income countr* OR middle income nation* OR low income countr* OR low income nation* OR poor countr* OR poor nation* OR lmic OR lmics Africa OR asia OR south america OR latin america OR central america OR Afghanistan*OR Albania* OR Algeria*OR Samoa* OR Angola* OR Armenia* OR Azerbaijan* OR Bangladesh* OR Bengali OR Belarus* OR Belize OR Benin OR Bhutan* OR Bolivia* OR Bosnia* OR Herzegovina* OR Botswana* OR Brazil* OR Bulgaria* OR Burkina Faso OR Burundi* OR Cabo Verd* OR Cape Verd* OR Cambodia* OR Cameroon* OR Central African* OR Chad* OR China OR Chinese OR Colombia* OR Comoros OR Congo OR Costa Rica* OR Cote d'Ivoire OR Ivory Coast OR Cuba OR Cuban OR Djibouti OR Dominica* OR Ecuador OR Egypt OR El Salvador* OR Eritrea* OR Ethiopia* OR Fiji* OR Gabon* OR Gambia* OR Georgia* OR Ghana* OR Grenada* OR Guatemala* OR Guinea* OR Guyan* OR Haiti* OR Hondura* OR India OR Indian* OR Indonesia* OR Iran* OR Iraq* OR Jamaica* OR Jordan* OR Kazakh* OR Kenya* OR Kiribati OR People's Republic of Korea OR North Korea OR Kosovo OR Kosovar* OR Kyrgyz* OR Lao OR Laos OR Laotian* OR Lebanon OR Lebanes* OR Lesotho OR Liberia* OR Libya* OR Macedonia* OR Madagascar* OR Malawi* OR Malaysia* OR Maldives OR Mali OR Marshall Island* OR Mauritania* OR Mauriti* OR Mexico OR Mexican* OR Micronesia* OR Moldova* OR Mongolia* OR Montenegr* OR Morocc* OR Mozambique OR Myanmar OR Burmese* OR Burma OR Namibia* OR Nauru OR Nepal* OR Nicaragua* OR Niger* OR Pakistan* OR Papua New Guinea OR Paraguay* OR Peru* OR Philippin* OR Romania* OR Russian Federation OR Rwanda* OR Samoa* OR Sao Tome OR Principe OR Senegal* OR Serbia* OR Sierra Leone* OR Solomon Island* OR Somalia* OR South Africa* OR South Sudan OR Sri Lanka OR St Lucia OR Saint Lucia OR St Vincent OR Saint Vincent OR Grenadines OR Sudan* OR Suriname* OR Swaziland* OR Syria* OR Tajik* OR Tanzania* OR Thai* OR Timor* OR Togo* OR Tonga* OR Tunisia* OR Turkey OR Turkish OR Turkmen* OR Tuvalu* OR Uganda* OR Ukrain* OR Uzbeki* OR Vanuatu* OR Vietnam* OR Viet nam* OR West Bank OR Gaza* OR Palestin* OR Yemen* OR Zambia* OR Zimbabw* |
|  | Mesh terms | "Developing Countries"[mesh] OR "Africa"[mesh] OR "Asia"[mesh] OR "South America"[mesh] OR "Latin America"[mesh] OR "Central America"[mesh] |
|  | Emtree terms | exp Developing Countries/ OR exp Africa/ OR exp Asia/ OR exp South America/ OR exp Latin America/ OR exp Central America/ |
|  | PsycINFO | exp Developing Countries/ OR exp Africa/ OR exp Asia/ OR exp South America/ OR exp Latin America/ OR exp Central America/ |
| Final search | Big term 1 AND Big term 2 AND Big term 3 AND Big term 4 | |

Title: Assessing cognition in people with severe mental disorders in low-and middle-income countries: a systematic review of assessment measures

Short title: Measures of cognition in severe mental disorders

Yohannes Gebreegziabhere^1, 2*^, Kassahun Habatmu^3^, Andualem Derese^2, 4^, Hetta Gouse^5^, Stephen M Lawrie^6^, Matteo Cella^7^, Atalay Alem^2^

^1^Department of Nursing, College of Health Sciences, Debre Berhan University, Debre Berhan, Ethiopia

^2^Department of Psychiatry, College of Health Sciences, Addis Ababa University, Addis Ababa, Ethiopia

^3^School of Psychology, College of Education and Behavioral Studies, Addis Ababa University, Addis Ababa, Ethiopia

^4^Department of Public Health, College of Health Sciences, Haremaya University, Harar, Ethiopia

^5^Department of Psychiatry and Mental Health, University of Cape Town, Cape Town, South Africa

^6^Department of Psychiatry, University of Edinburgh, Edinburgh, Scotland, United Kingdom

^7^Department of Psychology, Institute of Psychiatry, Psychology and Neuroscience, King's College London, London, England, United Kingdom

^*^Correspondence: Yohannes Gebreegziabhere Haile, Department of Nursing, Debre Berhan University, Debre Berhan, Ethiopia & Department of Psychiatry, College of Health Sciences, Addis Ababa University, Addis Ababa Ethiopia;

Phone number: +251 9 130 596 40;

Email: [yohannes36@gmail.com](mailto:yohannes36@gmail.com); or [yohannes36@dbu.edu.et](mailto:yohannes36@dbu.edu.et)
