# Supplementary material for: Assessing cognition in people with severe mental disorders in low- and middle-income countries: a systematic review of assessment measures
Source: Soc Psychiatry Psychiatr Epidemiol. 2021 Jun 18;57(3):435–60. doi: 10.1007/s00127-021-02120-x (PMC8934327; doi:10.1007/s00127-021-02120-x)
Supplement: Supplementary file 3 — Supplementary file3 (DOCX 124 KB) [file 127_2021_2120_MOESM3_ESM.docx]

# List of excluded articles

Online resources 3: List of excluded articles with reason of exclusion.

| **Citation (Author and year)** | **Title** | **Reason for excluded** |
| --- | --- | --- |
| [1] | Korean Facial Emotion Recognition Tasks for Schizophrenia Research | Not from LMIC (it is in Seoul, South Korea) |
| [2] | Performance of the Continuous Performance Test Among Community Samples | Not from LMIC (Chinshan, Taiwan, Chania) |
| [3] | Interview-based assessment of cognition is a strong predictor of quality of life in patients with schizophrenia and severe negative symptoms | Not a validation study (it is an association of cognitive measure with quality of life) |
| [4] | A qualitative assessment of cross-cultural adaptation of intermediate measures for schizophrenia in multisite  international studies | Not a validation study (it is a qualitative study of challenges of adaptation) |
| [5] | Brief Assessment of Cognition in Schizophrenia: Validation of the Japanese version | Not from LMIC (it is in Tokushima, Japan) |
| [6] | New instrument for measuring multiple domains of social cognition: Construct validity of the Social Cognition Screening Questionnaire (Japanese version) | Not from LMIC (it is in Teikyo, Japan) |
| [7] | The Portuguese version of the Clinical Global Impression – Schizophrenia Scale: validation study | Didn't address at list one domain (not at all about cognitive impairment) |
| [8] | Cross-cultural comparisons on Wisconsin Card Sorting Test performance in euthymic patients with bipolar disorder | Not from LMIC (Taiwan, Chaina, And US) |
| [9] | Measuring Cognitive Errors Using the Cognitive Distortions Scale (CDS): Psychometric Properties in Clinical and Non-Clinical Samples | Didn't address at list one domain (not at all about cognitive impairment) |
| [10] | Portuguese adaptation of the Cambridge Cognitive Examination-Revised in a  public geriatric outpatient clinic | None-English (May be Portuguese) |
| [11] | Revised Reading the Mind in the Eyes Test (RMET) – Brazilian version | PWSMD is not adequately represented it is an adaptation study among HC |
| [12] | Translation and cross-cultural adaptation of a mental health battery in an African setting | Not a validation study (it is a qualitative study of process of adaptation) |
| [13] | Reliability and validity of the California Verbal Learning Test-II – Japanese version | Not from LMIC (may be it is in Teikyo, Japan) |
| [14] | Validity and reliability of the Cognitive Complaints in Bipolar Disorder Rating Assessment (COBRA) in Japanese patients with bipolar disorder | Not from LMIC ( it is in Hokkaido, Japan) |
| [15] | Validation of the Chinese version of Brief Assessment of Cognition in Schizophrenia | Not from LMIC (it is in Chang Gung Memorial hospital, Taiwan, China) |
| [16] | Validity of the Montreal Cognitive Assessment Scale (MoCA) to detect cognitive impairment in schizophrenia | None-English (May be Spanish) |
| [17] | Reliability and validity of Turkish version of biological rhythms interview of assessment in neuropsychiatry | Not an article it is a poster presentation at 5th Biennial Conference of the International Society for Bipolar Disorders |
| [18] | The repeatable battery for the assessment of Neuropsychological status (RBANS) in patients with Schizophrenia: a preliminary study in Armenia | Not an article it is a poster presentation at 2nd Schizophrenia International Research Society Conference |
| [19] | Validity and Reliability of the Persian Language Version of the Neuropsychiatry Unit Cognitive Assessment Tool | PWSMD is not adequately represented the tool is designed for dementia than SMD |
| [20] | Adaptation of Protocole MEC de Poche and of the Extended MAC Battery: Brief MAC Battery | None-English (May be Portuguese) |
| [21] | Validation of the Cognitive Impairment in Psychiatry (SCIP-S) Screen Scale in Patients with Bipolar Disorder I | None-English (May be Spanish) |
| [22] | Self-perceived cognitive functioning and its relationship with objective performance in first episode schizophrenia: The Subjective Cognitive Impairment Scale | Not from LMIC (it is in Hong Kong, China) |
| [23] | Development and Psychometric Properties of the Taiwan Odd–Even Number Sequencing Test: A Nonalphabetic Measure of Working Memory | Not from LMIC (it is in Taiwan, China) |
| [24] | Performance of a Brazilian sample on the computerized Wisconsin Card Sorting Test | Not in PWSMD it is a normative data among students |
| [25] | Validation of the Brazilian version of mini-test CASI-S | Not in PWSMD it is in people with Alzheimer’s disease |
| [26] | Brazilian version of the Schizophrenia Cognition Rating Scale (SCoRS-Br) – Validation in clinical settings without informants | None-English (May be Portuguese) |
| [27] | Tunisian adaptation of Hopkins Verbal Learning Test , Form 1 | None-English (May be French) |
| [28] | Neuropsychological assessment of Mild cognitive impairment, Dementia  And Psychiatric disorder | Not an article it is a poster presentation at Alzheimer's Association International Conference 2016. Canada |
| [29] | Measuring theory of mind in schizophrenia research: Cross-cultural validation | Not from LMIC (it is in North America, and Korea (may be south Korea)) |
| [30] | The Tunisian cognitive battery for patients with schizophrenia | None-English (May be French) |
| [31] | Validity of the Chinese Version of the Allen Cognitive Level Screen Assessment for Individuals With Schizophrenia | Not from LMIC (it is in Honk Kong China) |
| [32] | Comparison of pervasive developmental disorder and schizophrenia by the Japanese version of the National Adult Reading Test | Not from LMIC (it is may be from Japan) |
| [33] | Validation of the Cognitive Impairment in Psychiatry (SCIP-S) Screen Scale in Patients with Bipolar Disorder I. | None-English (May be Spanish) |
| [34] | The Continuous Performance Test, Identical Pairs: norms, reliability and  performance in healthy controls and patients with schizophrenia in Singapore | Not from LMIC (it is may be from Singapore) |
| [35] | Neuropsychological diagnostics in Ethiopia - challenges and chances among Considerations regarding differential diagnosis (literature overview) | Not a validation study (it is a qualitative study of process of adaptation) |
| [36] | Development of brief versions of the Wechsler Intelligence Scale for schizophrenia: Considerations of the structure and predictability of intelligence | Not from LMIC (it is from Tokyo, Japan) |
| [37] | Validity of the Allen Cognitive Levels Assessment: a tri-ethnic comparison | Not from LMIC (it is from Texas, US) |
| [38] | Detecting cognitive impairment in patients with schizophrenia with the Addenbrooke’s Cognitive Examination | Not a validation study (it is a comparison of cognitive performance between PWS and HC) |
| [39] | "The Tunisian cognitive battery for patients with schizophrenia. (French)." | None-English (May be French) |
| [40] | Reliability and validity of the Brief Assessment of Cognition in Schizophrenia | None-English (May be Chinese) |
| [41] | Comparison between the results of the CISA and the WAIS-RC in patients with schizophrenia | None-English (May be Chinese) |
| [42] | Comparison of two measures assessing prospective memory impairment in elderly people with schizophrenia | None-English (May be Chinese) |
| [43] | Validity of the WAIS-RC short form in psychiatric inpatients | None-English (May be Chinese) |
| [44] | Reliability and Validity of Computerized Chinese Version of Cambridge Prospective Memory Test in Chronic Schizophrenia | None-English (May be Chinese) |
| [45] | The reliability and validity of schizophrenia cognition rating scale (Chinese version) | None-English (May be Chinese) |
| [46] | Stability of commonly used measures in the Wisconsin Card Sorting Test | None-English (May be Chinese) |
| [47] | Cognitive Function of Patients with Schizophrenia: Test of a Neuropsychological Training System | None-English (May be Chinese) |
| **Backward Search** | | |
| [48] | Applicability of the Rey Auditory-Verbal Learning Test to an adult sample in Brazil | Not in PWSMD it is a feasibility study among university students |
| [49] | Development of a Korean Version of the Perceived Deficits Questionnaire-Depression for Patients with Major Depressive Disorder | Not from LMIC (it is from South Korea) |
| [50] | Evaluation of two brief and Reliable estimates of the WAIS-R | Not from LMIC (it is may be from US) |
| [51] | Utility of WAIS-R short forms in schizophrenia | Not from LMIC (it is from Pittsburgh US) |
| [52] | Validity of Two Selected-Item Short Forms of the WAIS-III in an Intellectually Deficient Sample | Not in PWSMD and Not from LMIC |
| [53] | Concurrent validity of Three W\IS-R Short Forms in Psychiatric Inpatients | Not from LMIC (it is from Maryland, US) |
| [54] | Short form of the WAIS-III for use with patients with schizophrenia | Not from LMIC (it is from Maryland, US) |
| [55] | The Cognitive Failures Questionnaire (CFQ) and its correlates | Not from LMIC (it is from US) |
| [56] | The Schizophrenia Cognition Rating Scale: Validation of an interview-based  assessment of cognitive functioning in Asian patients with schizophrenia | Not from LMIC (it is from Singapore) |
| [57] | Discriminant and Factor Analysis of the WAIS and the Satz-Mogel Abbreviated WAIS on Brain-Damaged and Psychiatric Patients | Not in PWSMD (says bran damage and psychiatry patients doesn’t specify) |
| [58] | Factor analysis of the Frankfurt Complaint Questionnaire in a Spanish sample | Not from LMIC (it is from Pamplona, Spain) |
| [59] | Test–retest reliability and practice effects of Expanded Halstead–Reitan Neuropsychological Test Battery | Not in PWSMD (it is in HC) and is not from LMIC, is from US |
| [60] | Estimating the Full-Scale Score on the Wechsler Adult Intelligence Scale from Scores on Four Subtests | Not in PWSMD (it is in HC) and is not from LMIC, is from US |
| [61] | Comparison of the K-BIT with Short Forms of the WAIS-R in a Neuropsychological Population | Not from LMIC (it is from New Jersey, US) |
| [62] | Comparative validity of three Wechsler short forms for delinquents | Not in PWSMD (it is in HC from prison) and is not from LMIC, is from US |
| [63] | Optimising screening for cognitive dysfunction in bipolar disorder: Validation and evaluation of objective and subjective tools | Not from LMIC (it is from Copenhagen, Denmark) |
| [64] | The Brief Assessment of Cognition in Schizophrenia: reliability, sensitivity, and comparison with a standard neurocognitive battery | Not from LMIC (it is from three hospitals in US) |
| [65] | The Relationship of the Brief Assessment of Cognition in Schizophrenia (BACS) to Functional Capacity and Real-world Functional Outcome | Not from LMIC (it is from North Carolina in US) |
| [66] | Cognitive Screening Scale for Schizophrenia (CSSS): Part 1. Design and structure of the scale | Not from LMIC (it is from Warsaw, Poland) |
| [67] | Screening for cognitive dysfunction in unipolar depression: Validation  and evaluation of objective and subjective tools | Not from LMIC (it is from Copenhagen, Denmark) |
| [68] | Validity and reliability of a rating scale on subjective cognitive deficits in bipolar disorder (COBRA) | Not from LMIC (it is from Barcelona, Spain) |
| [69] | A brief cognitive assessment for use with schizophrenia patients in community clinics | Not from LMIC (it is from San Antonio State Hospital, US) |
| [70] | The Clock Drawing Test as a Measure of Executive Dysfunction in Elderly Depressed Patients | Not from LMIC (it is from California, US) |
| [71] | Investigating reliability of Reading Mind in the Eyes Test in a Turkish  population | Not in PWSMD (it is in HC (non-clinical population) |
| **Forward search** | | |
| [72] | Comprehensive review of the research employing the schizophrenia cognition rating scale (SCoRS) | Not a validation study (it is a review of ScoR) |
| [73] | Adaptation and validation of the Chinese version of the modified Leiden index of depression sensitivity | Didn't address at list one domain (not at all about cognitive impairment, it is about cognitive reactivity) |
| [74] | Validity of the Reading the Mind in the Eyes Test in a Brazilian Sample | Not in PWSMD (it is in HC (non-clinical population) |
| [75] | Croatian Adaptation of the Revised Reading the Mind in the Eyes Test (RMET) | Not from LMIC (it is from Croatia)  And  Not in PWSMD (it is in HC (non-clinical population) |
| [76] | Psychometric validation of the Perceived Deficits Questionnaire-Depression (PDQ-D) instrument in US and UK respondents with major depressive disorder | Not from LMIC (it is from US and UK) |
| [77] | Identifying cognitive subgroups in bipolar disorder: A cluster analysis | Not a validation study (it is a kind of cross-sectional study) |
| [78] | Expert Consensus on Screening and Assessment of Cognition in Psychiatry | Not a validation study (it is a kind of review and recommendation) |
| [79] | Assessing and addressing cognitive impairment in bipolar disorder: the International Society for Bipolar Disorders Targeting Cognition Task Force recommendations for clinicians | Not a validation study (it is a kind of review and recommendation) |
| [80] | Assessing Executive Dysfunction in  Neurodegenerative Disorders: A Critical Review of Brief Neuropsychological Tools | Not a validation study (it is a kind of review and recommendation) |
| [81] | Equivalence and practice effect of alternate forms For Malay version of Auditory Verbal Learning Test (MAVLT) | Not in PWSMD (it is in HC (non-clinical population) |
| [82] | Demographic and clinical factors associated with Verbal memory performance in patients with Schizophrenia in Hospital Universiti Sains Malaysia (HUSM), Malaysia | Not a validation study (it is a an association study) |
| [83] | Validation of the Malay Version of Short Informant Questionnaire on Cognitive Decline in the Elderly (MS-IQCODE) | Not in PWSMD (it is among attendants of post Stroke) |
| [84] | A systematic review of validity procedures used in neuropsychological batteries | Not a validation study (it is a review of validation procedures) |
| [85] | The Reading the Mind in the Eyes Test: A Portuguese version of the adults’ test | Not in PWSMD (it is in HC (non-clinical population) |
| [86] | Convergent validity of the Brazilian version of the Theory of Mind Task Battery for the assessment of social cognition in older adults | Not in PWSMD (it is in HC (non-clinical older population) |
| [87] | The Chinese Version of the Brief Assessment of Cognition in Schizophrenia: Data of a Large-Scale Mandarin-Speaking Population | Not in PWSMD (it is in HC (non-clinical population) and this study is from Taiwan |
| [88] | Validation of the Chinese version of Brief Assessment of Cognition in Schizophrenia | Not from LMIC (it is from Taiwan, China) |
| [89] | "Faux-Pas Test: A Proposal of a Standardized Short Version. | Full-text not found, author contacted and no response |
| **Backward search after forward and backward search included** | | |
| [90] | A comparison of the CogState Schizophrenia Battery and the Measurement and Treatment Research to Improve Cognition in Schizophrenia (MATRICS) Battery in assessing cognitive impairment in chronic schizophrenia | Not from LMIC (it is from North America, Australia, and the United Kingdom.) |
| [91] | Sensitivity and specificity of WAIS–III0WMS–III demographically corrected factor scores in neuropsychological assessment | Not from LMIC (it is from North America) in addition PWS are under represented |
| [92] | The evaluation of mentalization deficit with faux pas tests in schizophrenia | Not from LMIC (it is from Hungary) in addition there is evidence that it is non-English study |

# Reference (for List of excluded articles)

1. Bahk YC, Jang SK, Lee JY, Choi KH (2015) Korean facial emotion recognition tasks for schizophrenia research. Psychiatry Investigation 12 (2):235-241

2. Chen WJ, Hsiao CK, Hsiao LL, Hwu HG (1998) Performance of the Continuous Performance Test among community samples. Schizophrenia bulletin 24 (1):163-174. doi:10.1093/oxfordjournals.schbul.a033308

3. Cruz BF, Resende CBd, Carvalhaes CF, Cardoso CS, Teixeira AL, Keefe RS, Rocha FL, Salgado JV (2016) Interview-based assessment of cognition is a strong predictor of quality of life in patients with schizophrenia and severe negative symptoms. Rev bras psiquiatr 38 (3):216-221

4. Gonzalez JM, Rubin M, Fredrick MM, Velligan DI (2013) A qualitative assessment of cross-cultural adaptation of intermediate measures for schizophrenia in multisite international studies. Psychiatry research 206 (2-3):166-172. doi:10.1016/j.psychres.2012.10.015

5. Kaneda Y, Sumiyoshi T, Keefe R, Ishimoto Y, Numata S, Ohmori T (2007) Brief assessment of cognition in schizophrenia: validation of the Japanese version. Psychiatry and clinical neurosciences 61 (6):602-609. doi:10.1111/j.1440-1819.2007.01725.x

6. Kanie A, Hagiya K, Ashida S, Pu S, Kaneko K, Mogami T, Oshima S, Motoya M, Niwa S, Inagaki A, Ikebuchi E, Kikuchi A, Yamasaki S, Iwata K, Roberts DL, Nakagome K (2014) New instrument for measuring multiple domains of social cognition: construct validity of the Social Cognition Screening Questionnaire (Japanese version). Psychiatry and clinical neurosciences 68 (9):701-711. doi:10.1111/pcn.12181

7. Lima MSd, Soares BGdO, Paoliello G, Vieira RM, Martins CM, Mota Neto JId, Ferrão Y, Schirmer DA, Volpe FM (2007) The Portuguese version of the Clinical Global Impression - Schizophrenia Scale: validation study. Rev bras psiquiatr 29 (3):246-249

8. Liu YM, Tsai SY, Fleck DE, Strakowski SM (2011) Cross-cultural comparisons on Wisconsin Card Sorting Test performance in euthymic patients with bipolar disorder. Psychiatry research 189 (3):469-471. doi:10.1016/j.psychres.2011.05.038

9. Ozdel K, Taymur I, Guriz SO, Tulaci RG, Kuru E, Turkcapar MH (2014) Measuring cognitive errors using the Cognitive Distortions Scale (CDS): psychometric properties in clinical and non-clinical samples. PloS one 9 (8):e105956. doi:10.1371/journal.pone.0105956

10. Paradela EMP, Lopes CdS, Lourenço RA (2009) Adaptação para o português do Cambridge Cognitive Examination-Revised aplicado em um ambulatório público de geriatria (Portuguese adaptation of the Cambridge Cognitive Examination-Revised in a public geriatric outpatient clinic). Cad saúde pública 25 (12):2562-2570

11. Sanvicente-Vieira B, Kluwe-Schiavon B, Wearick-Silva LE, Piccoli GL, Scherer L, Tonelli HA, Grassi-Oliveira R (2014) Revised Reading the Mind in the Eyes Test (RMET) - Brazilian version. Rev bras psiquiatr 36 (1):60-67

12. Smit J, van den Berg CE, Bekker LG, Seedat S, Stein DJ (2006) Translation and cross-cultural adaptation of a mental health battery in an African setting. African Health Sciences 6 (4):215-222

13. Sumiyoshi T, Toyomaki A, Kawano N, Kitajima T, Kusumi I, Ozaki N, Iwata N, Nakagome K (2017) Reliability and validity of the California Verbal Learning Test-II - Japanese version. Psychiatry and clinical neurosciences 71 (6):417-418. doi:10.1111/pcn.12525

14. Toyoshima K, Fujii Y, Mitsui N, Kako Y, Asakura S, Martinez-Aran A, Vieta E, Kusumi I (2017) Validity and reliability of the Cognitive Complaints in Bipolar Disorder Rating Assessment (COBRA) in Japanese patients with bipolar disorder. Psychiatry research 254:85-89. doi:10.1016/j.psychres.2017.04.043

15. Wang LJ, Lin PY, Lee Y, Huang YC, Hsu ST, Hung CF, Chen CK, Chen YC, Wang YL, Tsai MC (2016) Validation of the Chinese version of Brief Assessment of Cognition in Schizophrenia. Neuropsychiatr Dis Treat 12:2819-2826. doi:10.2147/ndt.s118110

16. Anon (2014) Validez de la Escala de Evaluación Cognitiva de Montreal (MoCA) para determinar deterioro cognitivo en pacientes con esquizofrenia (Validity of the Montreal Cognitive Assessment Scale (MoCA) to detect cognitive impairment in schizophrenia). Salud ment 37 (6):517-522

17. Aydemir O, Akkaya C, Altinbas K, Kora K, Suculluoglu Dikici D, Akdeniz F, Kalayci F, Oral ET, Vahip S (2012) Reliability and validity of Turkish version of biological rhythms interview of assessment in neuropsychiatry. Bipolar disorders 1):57

18. Azizian A, Yeghiyan M, Ishkhanyan B (2010) The Repeatable Battery for the Assessment of Neuropsychological Status (RBANS) in patients with schizophrenia: A preliminary study in Armenia. Schizophrenia research 117 (2-3):206

19. Barekatain M, Walterfang M, Behdad M, Tavakkoli M, Mahvari J, Maracy MR, Velakoulis D (2010) Validity and reliability of the Persian language version of the neuropsychiatry unit cognitive assessment tool. Dementia and geriatric cognitive disorders 29 (6):516-522. doi:10.1159/000313981

20. Casarin FS, Scherer LC, Ferré P, Ska B, Parente MAPdM, Joanette Y, Fonseca RP (2013) Adaptação do Protocole MEC de Poche e da Bateria Mac Expandida: Bateria Mac Breve (Adaptation of Protocole MEC de Poche and of the Extended MAC Battery: Brief MAC Battery

Adaptación del protocole MEC Poche y de la Batería MAC Ampliada: Batería MAC Breve). Psico (Porto Alegre) 44 (2):288-299

21. Castano Ramirez OM, Martinez Ramirez YA, Marulanda Mejia F, Diaz Cabezas R, Valderrama Sanchez LA, Varela Cifuentes V, Aguirre Acevedo DC (2015) [Validation of the Cognitive Impairment in Psychiatry (SCIP-S) Screen Scale in Patients with Bipolar Disorder I]. Revista colombiana de psiquiatria 44 (1):20-27. doi:10.1016/j.rcp.2014.08.002

22. Chang WC, Chan TCW, Chiu SS, Hui CLM, Chan SKW, Lee EHM, Chen EYH (2015) Self-perceived cognitive functioning and its relationship with objective performance in first-episode schizophrenia: The Subjective Cognitive Impairment Scale. Comprehensive psychiatry 56:42-50

23. Chen YT, Peng CY, Hua MS, Liu CC, Chen HY, Hwu HG (2018) Development and Psychometric Properties of the Taiwan Odd-Even Number Sequencing Test: A Nonalphabetic Measure of Working Memory. Assessment 25 (2):183-192

24. Coelho LF, Rosário MCd, Mastrorosa RS, Miranda MC, Bueno OFA (2012) Performance of a Brazilian sample on the computerized Wisconsin Card Sorting Test. Psychol neurosci (Impr) 5 (2):147-156

25. Damasceno A, Delicio AM, Mazo DFC, Zullo JFD, Scherer P, Ng RTY, Damasceno BP (2005) Validation of the Brazilian version of mini-test CASI-S. Arq neuropsiquiatr 63 (2b)

26. de Castro Ferreira Junior B, do Amaral Barbosa M, Barbosa IG, Borges A, Hara C, Rocha FL (2010) Brazilian version of the Schizofrenio Cognition Rating Scale (SCoRS-Br) - Validation in clinical settings without informants. Jornal Brasileiro de Psiquiatria 59 (4):271-278

27. Dellagi L, Ben Azouz O, Johnson I, Kebir O, Amado I, Tabbane K (2009) [Tunisian adaptation of Hopkins Verbal Learning Test , Form 1]. La Tunisie medicale 87 (10):670-673

28. Karakoc E, Cangoz B, Selekler K (2016) Neuropsychological assessment of mild cognitive impairment, dementia and psychiatric disorder. Alzheimer's and Dementia 12 (7 Supplement):P965

29. Lee HS, Poltorak A, Park K, Assaf M, Bell M, Wexler B, Choi KH, Corbera S, Lee H (2017) Measuring implicit theory of mind in schizophrenia research: Cross-cultural validation. Schizophrenia bulletin 43 (Supplement 1):S171

30. Olfa BA, Lamia D, Oussama K, Ines J, Isabelle m, Karim T (2009) [&lt;The&gt; tunisian cognitive battery for patients with schizophrenia]. Tunisie Med 87 (10):674-679

31. Leung SB, Man DW (2007) Validity of the Chinese Version of the Allen Cognitive Level Screen Assessment for Individuals With Schizophrenia. OTJR: Occupation, Participation and Health 27 (1):31-40

32. Ota T, Iida J, Sawada M, Suehiro Y, Kishimoto N, Tanaka S, Nagauchi K, Nakanishi Y, Yamamuro K, Negoro H, Iwasaka H, Sadamatsu M, Kishimoto T (2013) Comparison of pervasive developmental disorder and schizophrenia by the Japanese version of the National Adult Reading Test. International journal of psychiatry in clinical practice 17 (1):10-15. doi:10.3109/13651501.2011.653380

33. Ramirez OMC, Ramirez YAM, Mejia FM, Cabezas RD, Sanchez LAV, Cifuentes VV, Acevedo DCA (2015) Validation of the Cognitive Impairment in Psychiatry (SCIP-S) Screen Scale in Patients with Bipolar Disorder I. Revista colombiana de psiquiatria 44 (1):20-27

34. Rapisarda A, Kraus M, Tan YW, Lam M, Eng GK, Lee J, Subramaniam M, Collinson SL, Chong SA, Keefe RSE (2014) The continuous performance test, identical pairs: Norms, reliability and performance in healthy controls and patients with schizophrenia in Singapore. Schizophrenia research 156 (2-3):233-240

35. Steinboeck C (2015) Neuropsychological Diagnostics in Ethiopia - Challenges and Chances among Considerations Regarding Differential Diagnosis (Literature Overview). African Journal of Neurological Sciences

36. Sumiyoshi C, Uetsuki M, Suga M, Kasai K, Sumiyoshi T (2013) Development of brief versions of the wechsler intelligence scale for schizophrenia. Schizophrenia bulletin 1):S276

37. Velligan DI, True JE, Lefton RS, Moore TC, Flores CV (1995) Validity of the Allen Cognitive Levels Assessment: A tri-ethnic comparison. Psychiatry research 56 (2):101-109

38. Charernboon T, Chompookard P (2019) Detecting cognitive impairment in patients with schizophrenia with the Addenbrooke's Cognitive Examination. Asian journal of psychiatry 40:19-22. doi:10.1016/j.ajp.2019.01.006

39. Ben Azouz O, Dellagi L, Kebir O, Johnson I, Amado I, Tabbane K (2009) The Tunisian cognitive battery for patients with schizophrenia. [French] (La batterie Tunisienne d'Evaluation cognitive dans la schizophrenie.). Tunisie Medicale 87 (10):674-679

40. Cui J-F, Zou Y-Z, Li Y, Wang J, Chen N, Fan H-Z, Zhang D, Xu Z, Song S-G, Wang Y-H, Gao W-J, Duan J-H (2009) Reliability and validity of the Brief Assessment of Cognition in Schizophrenia. Chinese Mental Health Journal 23 (3):183-187

41. Dong H, Lu Y, Zhang Q, Li X (2003) Comparison between the results of the CISA and the WAIS-RC in patients with schizophrenia. Chinese Mental Health Journal 17 (3):178

42. Gao X-X, Zou Y-Z, Chen N, Fan H-Z, Wen Y-J, Liu L-L (2015) Comparison of two measures assessing prospective memory impairment in elderly people with schizophrenia. Chinese Mental Health Journal 29 (7):516-521

43. Jiang C, Liu J, Zhang X (1999) Validity of the WAIS-RC short form in psychiatric inpatients. Chinese Journal of Clinical Psychology 7 (2):74-76

44. Mengjie XIE, Yizhuang ZOU, Zhiren W, Nan C, Yong Z, Xiaoxiao GAO, Wenjing Z (2014) Reliability and Validity of Computerized Chinese Version of Cambridge Prospective Memory Test in Chronic Schizophrenia (中国康复理论与实践). Chinese Journal of Rehabilitation Theory and Practice:950-954

45. Shisheng HAO, Huiling W, Jingjing Z, Xuan LIU, Gaohua W (2011) The reliability and validity of schizophrenia cognition rating scale (Chinese version) (中华行为医学与脑科学杂志). Chinese Journal of Behavioral Medicine and Brain Science:564-567

46. Tan Y, Zou Y, Qu Y, Guo X (2002) Stability of commonly used measures in the Wisconsin Card Sorting Test. Chinese Mental Health Journal 16 (12):831-833

47. Xiao Q, Wang C, Yin W-G (2005) Cognitive Function of Patients with Schizophrenia: Test of a Neuropsychological Training System. Chinese Mental Health Journal 19 (9):595-599

48. Salgado JV, Malloy-Diniz LF, Abrantes SS, Moreira L, Schlottfeldt CG, Guimaraes W, Freitas DM, Oliveira J, Fuentes D (2011) Applicability of the Rey auditory-verbal learning test to an adult sample in Brazil. Braz J Psychiatry 33 (3):234-237

49. Kim JM, Hong JP, Kim SD, Kang HJ, Lee YS (2016) Development of a Korean Version of the Perceived Deficits Questionnaire-Depression for Patients with Major Depressive Disorder. Clinical psychopharmacology and neuroscience : the official scientific journal of the Korean College of Neuropsychopharmacology 14 (1):26-32. doi:10.9758/cpn.2016.14.1.26

50. Axelrod BN, Naugle RI (1998) Evaluation of two brief and reliable estimates of the WAIS-R. The International journal of neuroscience 94 (1-2):85-91. doi:10.3109/00207459808986441

51. Allen DN, Huegel SG, Gurklis JA, Jr., Kelley ME, Barry EJ, van Kammen DP (1997) Utility of WAIS-R short forms in schizophrenia. Schizophrenia research 26 (2-3):163-172

52. Alley PJ, Allen RA, Leverett JP (2007) Validity of two selected-item short forms of the WAIS-III in an intellectually deficient sample. Journal of clinical psychology 63 (12):1145-1152. doi:10.1002/jclp.20410

53. Benedict RH, Schretlen D, Bobholz JH (1992) Concurrent validity of three WAIS—R short forms in psychiatric inpatients. Psychological assessment 4 (3):322

54. Blyler CR, Gold JM, Iannone VN, Buchanan RW (2000) Short form of the WAIS-III for use with patients with schizophrenia. Schizophrenia research 46 (2-3):209-215

55. Broadbent DE, Cooper PF, FitzGerald P, Parkes KR (1982) The Cognitive Failures Questionnaire (CFQ) and its correlates. The British journal of clinical psychology 21 (Pt 1):1-16

56. Chia MY, Chan WY, Chua KY, Lee H, Lee J, Lee R, Lim C, Tay E, Woon PS, Keefe RS, Sim K (2010) The Schizophrenia Cognition Rating Scale: validation of an interview-based assessment of cognitive functioning in Asian patients with schizophrenia. Psychiatry research 178 (1):33-38. doi:10.1016/j.psychres.2010.03.020

57. Coolidge FL (1976) Discriminant and factor analysis of the WAIS and the Satz-Mogel Abbreviated WAIS on brain-damaged and psychiatric patients. Journal of consulting and clinical psychology 44 (1):153

58. Cuesta MJ, Peralta V, Irigoyen I (1996) Factor analysis of the Frankfurt Complaint Questionnaire in a Spanish sample. Psychopathology 29 (1):46-53. doi:10.1159/000284971

59. Dikmen SS, Heaton RK, Grant I, Temkin NR (1999) Test-retest reliability and practice effects of expanded Halstead-Reitan Neuropsychological Test Battery. Journal of the International Neuropsychological Society : JINS 5 (4):346-356

60. Doppelt JE (1956) Estimating the full scale score on the Wechsler Adult Intelligence Scale from scores on four subjects. Journal of Consulting Psychology 20 (1):63

61. Eisenstein N, Engelhart CI (1997) Comparison of the K--BIT with short forms of the WAIS--R in a neuropsychological population. Psychological assessment 9 (1):57

62. Haynes JP (1983) Comparative validity of three Wechsler short forms for delinquents. Journal of clinical psychology 39 (2):275-278. doi:10.1002/1097-4679(198303)39:2<275::aid-jclp2270390225>3.0.co;2-e

63. Jensen JH, Stottrup MM, Nayberg E, Knorr U, Ullum H, Purdon SE, Kessing LV, Miskowiak KW (2015) Optimising screening for cognitive dysfunction in bipolar disorder: Validation and evaluation of objective and subjective tools. Journal of affective disorders 187:10-19. doi:10.1016/j.jad.2015.07.039

64. Keefe RS, Goldberg TE, Harvey PD, Gold JM, Poe MP, Coughenour L (2004) The Brief Assessment of Cognition in Schizophrenia: reliability, sensitivity, and comparison with a standard neurocognitive battery. Schizophrenia research 68 (2-3):283-297. doi:10.1016/j.schres.2003.09.011

65. Keefe RS, Poe M, Walker TM, Harvey PD (2006) The relationship of the Brief Assessment of Cognition in Schizophrenia (BACS) to functional capacity and real-world functional outcome. Journal of clinical and experimental neuropsychology 28 (2):260-269. doi:10.1080/13803390500360539

66. Mosiolek A, Gierus J, Koweszko T, Borkowska A, Janus M, Szulc A (2018) The Cognitive Screening Scale for Schizophrenia (CSSS). Part 1. Design and structure of the scale. Psychiatria polska 52 (2):227-239. doi:10.12740/PP/OnlineFirst/64831

67. Ott CV, Bjertrup AJ, Jensen JH, Ullum H, Sjaelland R, Purdon SE, Vieta E, Kessing LV, Miskowiak KW (2016) Screening for cognitive dysfunction in unipolar depression: Validation and evaluation of objective and subjective tools. Journal of affective disorders 190:607-615. doi:10.1016/j.jad.2015.10.059

68. Rosa AR, Mercade C, Sanchez-Moreno J, Sole B, Mar Bonnin CD, Torrent C, Grande I, Sugranyes G, Popovic D, Salamero M, Kapczinski F, Vieta E, Martinez-Aran A (2013) Validity and reliability of a rating scale on subjective cognitive deficits in bipolar disorder (COBRA). Journal of affective disorders 150 (1):29-36. doi:10.1016/j.jad.2013.02.022

69. Velligan DI, DiCocco M, Bow-Thomas CC, Cadle C, Glahn DC, Miller AL, Biggs MM, Shores-Wilson K, McKenzie CA, Crismon ML (2004) A brief cognitive assessment for use with schizophrenia patients in community clinics. Schizophrenia research 71 (2-3):273-283. doi:10.1016/j.schres.2004.02.027

70. Woo BK, Rice VA, Legendre SA, Salmon DP, Jeste DV, Sewell DD (2004) The clock drawing test as a measure of executive dysfunction in elderly depressed patients. Journal of geriatric psychiatry and neurology 17 (4):190-194. doi:10.1177/0891988704269820

71. Yildirim EA, Kasar M, Guduk M, Ates E, Kucukparlak I, Ozalmete EO (2011) Investigation of the reliability of the "reading the mind in the eyes test" in a Turkish population. Turk psikiyatri dergisi = Turkish journal of psychiatry 22 (3):177-186

72. Harvey PD, Khan A, Atkins A, Walker TM, Keefe RS (2019) Comprehensive review of the research employing the schizophrenia cognition rating scale (SCoRS). Schizophrenia research

73. Huang FF, Li H, Lei Y, Li Q, Chen B (2019) Adaptation and validation of the Chinese version of the modified Leiden index of depression sensitivity. Journal of affective disorders

74. Koich Miguel F, Barquete Caramanico R, Yudi Huss E, Carolina Zuanazzi A (2017) Validity of the Reading the Mind in the Eyes Test in a Brazilian Sample. Paideia (0103863X) 27 (66)

75. Kotrla Topić M, Perković Kovačević M (2019) Croatian Adaptation of the Revised Reading the Mind in the Eyes Test (RMET). Psihologijske teme 28 (2):377-395

76. Lam RW, Lamy F-X, Danchenko N, Yarlas A, White MK, Rive B, Saragoussi D (2018) Psychometric validation of the Perceived Deficits Questionnaire-Depression (PDQ-D) instrument in Us and UK respondents with major depressive disorder. Neuropsychiatric disease and treatment 14:2861

77. Lima F, Rabelo-da-Ponte FD, Bücker J, Czepielewski L, Hasse-Sousa M, Telesca R, Solé B, Reinares M, Vieta E, Rosa AR (2019) Identifying cognitive subgroups in bipolar disorder: A cluster analysis. Journal of affective disorders 246:252-261

78. McIntyre RS, Anderson N, Baune BT, Brietzke E, Burdick K, Fossati P, Gorwood P, Harmer C, Harrison J, Harvey P (2019) Expert Consensus on Screening and Assessment of Cognition in Psychiatry. CNS spectrums 24 (1):154-162

79. Miskowiak K, Burdick K, Martinez‐Aran A, Bonnin C, Bowie C, Carvalho A, Gallagher P, Lafer B, López‐Jaramillo C, Sumiyoshi T (2018) Assessing and addressing cognitive impairment in bipolar disorder: the International Society for Bipolar Disorders Targeting Cognition Task Force recommendations for clinicians. Bipolar disorders 20 (3):184-194

80. Moreira HS, Costa AS, Castro SL, Lima CF, Vicente SG (2017) Assessing executive dysfunction in neurodegenerative disorders: a critical review of brief neuropsychological tools. Frontiers in aging neuroscience 9:369

81. Munjir N, Othman Z, Zakaria R, Shafin N, Hussain NA, Desa AM, Ahmad AH (2015) Equivalence and practice effect of alternate forms for Malay version of Auditory Verbal Learning Test (MAVLT). EXCLI journal 14:801

82. Othman Z, Jamaluddin R, Alwi MNM, Ismail HC (2011) Demographic and clinical factors associated with verbal memory performance in patients with schizophrenia in Hospital Universiti Sains Malaysia (HUSM), Malaysia. Advisory Board, Associate Editors, Reviewers and Editorial Board Members iii-v Information and Guideline for Authors vi:148

83. Othman Z, Wong ST, Drahman I, Wee KW (2015) Validation of the Malay Version of Short Informant Questionnaire on Cognitive Decline in the Elderly. International Medical Journal 22 (4):260-262

84. Pawlowski J, Segabinazi JD, Wagner F, Bandeira DR (2013) A systematic review of validity procedures used in neuropsychological batteries. Psychology & Neuroscience 6 (3):311

85. Pestana J, Menéres MSSPC, Gouveia MJPM, Oliveira RF (2018) The Reading the Mind in the Eyes Test: A Portuguese version of the adults’ test. Análise Psicológica 36 (3):369-381

86. Rezende TFd, Bomfim AJdL, Chagas NMdS, Osório FdL, Chagas MHN (2018) Convergent validity of the Brazilian version of the Theory of Mind Task Battery for the assessment of social cognition in older adults. Archives of Clinical Psychiatry (São Paulo) 45 (3):75-76

87. Wang L-J, Huang Y-C, Hung C-F, Chen C-K, Chen Y-C, Lee P-Y, Wang S-M, Liu M-H, Lin C-J, Hsu S-T (2016) The Chinese version of the brief assessment of cognition in schizophrenia: data of a large-scale mandarin-speaking population. Archives of Clinical Neuropsychology 32 (3):289-296

88. Wang L-J, Lin P-Y, Lee Y, Huang Y-C, Hsu S-T, Hung C-F, Chen C-K, Chen Y-C, Wang Y-L, Tsai M-C (2016) Validation of the Chinese version of Brief Assessment of Cognition in Schizophrenia. Neuropsychiatric disease and treatment 12:2819

89. Fernandez-Modamio M, Arrieta-Rodriguez M, Bengochea-Seco R, Santacoloma-Cabero I, Gomez de Tojeiro-Roce J, Garcia-Polavieja B, Gonzalez-Fraile E, Martin-Carrasco M, Griffin K, Gil-Sanz D (2018) Faux-Pas Test: A Proposal of a Standardized Short Version. Clinical schizophrenia & related psychoses. doi:10.3371/csrp.fear.061518

90. Pietrzak RH, Olver J, Norman T, Piskulic D, Maruff P, Snyder PJ (2009) A comparison of the CogState Schizophrenia Battery and the Measurement and Treatment Research to Improve Cognition in Schizophrenia (MATRICS) Battery in assessing cognitive impairment in chronic schizophrenia. Journal of clinical and experimental neuropsychology 31 (7):848-859. doi:10.1080/13803390802592458

91. Taylor MJ, Heaton RK (2001) Sensitivity and specificity of WAIS–III/WMS–III demographically corrected factor scores in neuropsychological assessment. Journal of the International Neuropsychological Society 7 (7):867-874

92. Varga E, Tenyi T, Fekete S, Herold R (2008) [The evaluation of mentalization deficit by the faux pas test in schizophrenia]. Neuropsychopharmacologia Hungarica : a Magyar Pszichofarmakologiai Egyesulet lapja = official journal of the Hungarian Association of Psychopharmacology 10 (2):75-80

Title: Assessing cognition in people with severe mental disorders in low-and middle-income countries: a systematic review of assessment measures

Short title: Measures of cognition in severe mental disorders

Yohannes Gebreegziabhere^1, 2*^, Kassahun Habatmu^3^, Andualem Derese^2, 4^, Hetta Gouse^5^, Stephen M Lawrie^6^, Matteo Cella^7^, Atalay Alem^2^

^1^Department of Nursing, College of Health Sciences, Debre Berhan University, Debre Berhan, Ethiopia

^2^Department of Psychiatry, College of Health Sciences, Addis Ababa University, Addis Ababa, Ethiopia

^3^School of Psychology, College of Education and Behavioral Studies, Addis Ababa University, Addis Ababa, Ethiopia

^4^Department of Public Health, College of Health Sciences, Haremaya University, Harar, Ethiopia

^5^Department of Psychiatry and Mental Health, University of Cape Town, Cape Town, South Africa

^6^Department of Psychiatry, University of Edinburgh, Edinburgh, Scotland, United Kingdom

^7^Department of Psychology, Institute of Psychiatry, Psychology and Neuroscience, King's College London, London, England, United Kingdom

^*^Correspondence: Yohannes Gebreegziabhere Haile, Department of Nursing, Debre Berhan University, Debre Berhan, Ethiopia & Department of Psychiatry, College of Health Sciences, Addis Ababa University, Addis Ababa Ethiopia;

Phone number: +251 9 130 596 40;

Email: [yohannes36@gmail.com](mailto:yohannes36@gmail.com); or [yohannes36@dbu.edu.et](mailto:yohannes36@dbu.edu.et)
