# Supplementary material for: Assessing cognition in people with severe mental disorders in low- and middle-income countries: a systematic review of assessment measures
Source: Soc Psychiatry Psychiatr Epidemiol. 2021 Jun 18;57(3):435–60. doi: 10.1007/s00127-021-02120-x (PMC8934327; doi:10.1007/s00127-021-02120-x)
Supplement: Supplementary file 4 — Supplementary file4 (DOCX 75 KB) [file 127_2021_2120_MOESM4_ESM.docx]

Online resource 4: Quality assessment of the included articles per each measurement properties

| **S.N** | **Citation (Author and year)** | **Name of the measure** | **Quality of the psychometric property assessed** | | | | | | | | | | | |
| --- | --- | --- | --- | --- | --- | --- | --- | --- | --- | --- | --- | --- | --- | --- |
|  |  |  | **Content validity** | | | | | **Structural validity** | **Internal consistency** | **Cross-cultural validity** | **Test-retest reliability** | **Criterion validity** | **Hypotheses testing** | |
|  |  |  | **Relevance (pt)** | **Comprehensiveness (pt)** | **Comprehensibility (pt)** | **Relevance (pr)** | **Comprehensiveness (pr)** |  |  |  |  |  | **Concurrent/Convergent validity** | **Discriminant validity** |
| 1 | (Araujo et al., 2015) (1) | BACS |  |  |  |  |  | A | V | D | D |  | V | V |
| 2 | (Salgado et al., 2007) (2) | BACS |  |  |  |  |  |  | V | D |  |  |  | V |
| 3 | (Mazhari et al., 2014) (3) | BACS |  |  |  |  |  | D | D | D |  | D | D | D |
| 4 | (Muliady et al., 2019) (4) | BACS | D | D | D | D | D |  | V | D | D |  | V |  |
| 5 | (Abdullah et al., 2013) (5) | BACS |  |  |  |  |  |  |  | D | A |  |  |  |
| 6 | (Azizian et al., 2011) (6) | RBANS | D | D | D |  |  |  | V | D | A |  | V | V |
| **Score:** V= very good; A = adequate; D = doubtful; I = inadequate; N= not applicable  **Abbreviation**: BACS: Brief Assessment of Cognition in Schizophrenia; pr: professional; pt: patients; RBANS; Repeatable Battery for the Assessment of Neuropsychological Status | | | | | | | | | | | | | | |
| **S.N** | **Citation (Author and year)** | **Name of the measure** | **Quality of the psychometric property assessed** | | | | | | | | | | | |
|  |  |  | **Content validity** | | | | | **Structural validity** | **Internal consistency** | **Cross-cultural validity** | **Test-retest reliability** | **Criterion validity** | **Hypotheses testing** | |
|  |  |  | **Relevance (pt)** | **Comprehensiveness (pt)** | **Comprehensibility (pt)** | **Relevance (pr)** | **Comprehensiveness (pr)** |  |  |  |  |  | **Concurrent/Convergent validity** | **Discriminant validity** |
| 7 | (Johnson et al., 2009) (7) | SASCCS |  |  |  |  |  | A | V |  | D |  | V |  |
| 8 | (Aydemir et al., 2017) (8) | PDQ-D & BCCCI |  |  |  |  |  | A | V | D |  |  | V | V |
| 9 | (Shi et al., 2017) (9) | PDQ-D |  |  |  |  |  | D | D |  | D |  | D | D |
| 10 | (Bosgelmez et al., 2015) (10) | CAI | D | D | D |  |  |  | V | D |  |  | V |  |
| 11 | (Changiz et al., 2011) (11) | WCST |  |  |  |  |  |  |  |  |  |  | V | V |
| 12 | (Dias et al., 2017) (12) | BCB |  |  |  |  |  |  |  |  |  |  | D | D |
| **Score:** V= very good; A = adequate; D = doubtful; I = inadequate; N= not applicable  **Abbreviation**: BCB: Brief cognitive battery; BCCCI: British Columbia Cognitive Complaints Inventory; CAI: Cognitive Assessment Interview; PDQ-D: Perceived Deficit Questionnaire-Depression; ; pr: professional; pt: patients; SASCCS: Self-Assessment Scale of Cognitive Complaints in Schizophrenia; WCST: The Wisconsin Test Card Sorting Test | | | | | | | | | | | | | | |
| **S.N** | **Citation (Author and year)** | **Name of the measure** | **Quality of the psychometric property assessed** | | | | | | | | | | | |
|  |  |  | **Content validity** | | | | | **Structural validity** | **Internal consistency** | **Cross-cultural validity** | **Test-retest reliability** | **Criterion validity** | **Hypotheses testing** | |
|  |  |  | **Relevance (pt)** | **Comprehensiveness (pt)** | **Comprehensibility (pt)** | **Relevance (pr)** | **Comprehensiveness (pr)** |  |  |  |  |  | **Concurrent/Convergent validity** | **Discriminant validity** |
| 13 | (Fan et al., 2019) (13) | SF4-WAIS-IV (FS) |  |  |  |  |  |  | V |  | D | V | V |  |
| 14 | (Fonseca et al., 2017) (14) | MCCB |  |  | D |  |  |  | V | D | D |  | V | V |
| 15 | (Mazhari et al., 2017) (15) | SCoRS |  |  |  |  |  |  |  | D |  |  | V | V |
| 16 | (Negrão et al., 2016) (16) | SV-FPRT |  |  |  | D | D |  | D |  |  |  |  | D |
| 17 | (Sanvicente-Vieira et al., 2012) (17) | The ToM Stories and the Hinting Task | D | D | D |  |  |  |  | D |  |  |  |  |
| **Score:** V= very good; A = adequate; D = doubtful; I = inadequate; N= not applicable  **Abbreviation**: MCCB: MATRICS Consensus Cognitive Battery; ; pr: professional; pt: patients; SCoRS: Schizophrenia Cognition Rating Scale; SF4-WAIS-IV (FS): The four-subtest index-based short form (SF4) of Wechsler Adult Intelligence Scale -IV full scale; SV-FPRT: Short version of the Faux Pas Recognition Test: ToM: Theory of Mind | | | | | | | | | | | | | | |
| **S.N** | **Citation (Author and year)** | **Name of the measure** | **Quality of the psychometric property assessed** | | | | | | | | | | | |
|  |  |  | **Content validity** | | | | | **Structural validity** | **Internal consistency** | **Cross-cultural validity** | **Test-retest reliability** | **Criterion validity** | **Hypotheses testing** | |
|  |  |  | **Relevance (pt)** | **Comprehensiveness (pt)** | **Comprehensibility (pt)** | **Relevance (pr)** | **Comprehensiveness (pr)** |  |  |  |  |  | **Concurrent/Convergent validity** | **Discriminant validity** |
| 18 | (Morozova et al., 2017) (18) | Hinting Task', 'Faux Pas', and RMET |  |  |  |  |  |  |  |  |  |  |  |  |
| 19 | (Pieters and Sieberhagen, 1986) (19) | SAWAIS- SF |  |  |  |  |  |  |  |  |  |  | V |  |
| 20 | (Ruzita et al., 2009) (20) | AVLT | D | D | D | D | D | I |  | D | D |  |  | V |
| 21 | (Tuncay et al., 2013) (21) | FAB | D | D | D |  |  |  | V | D | A |  | V | V |
| 22 | (Gulec et al., 2008) (22) | FAB |  |  |  |  |  |  | V |  | D |  | V | V |
| **Score:** V= very good; A = adequate; D = doubtful; I = inadequate; N= not applicable  **Abbreviation**: AVLT: Auditory verbal learning test; FAB: Frontal Assessment Battery:; pr: professional; pt: patients; RMET: Revised Reading the Mind in the Eyes Test; SAWAIS- SF: South African Wechsler Adult Intelligence Scale; SF: Short Form | | | | | | | | | | | | | | |
| **S.N** | **Citation (Author and year)** | **Name of the measure** | **Quality of the psychometric property assessed** | | | | | | | | | | | |
|  |  |  | **Content validity** | | | | | **Structural validity** | **Internal consistency** | **Cross-cultural validity** | **Test-retest reliability** | **Criterion validity** | **Hypotheses testing** | |
|  |  |  | **Relevance (pt)** | **Comprehensiveness (pt)** | **Comprehensibility (pt)** | **Relevance (pf)** | **Comprehensiveness (pf)** |  |  |  |  |  | **Concurrent/Convergent validity** | **Discriminant validity** |
| 23 | (Lima et al., 2018) (23) | COBRA |  |  |  |  |  | D | D | D |  | D | D | D |
| 24 | (Xiao et al., 2015)(24) | COBRA | D | D | D | D | D | V | V | D | A | V | V | V |
| 25 | (Yoldi-Negrete et al., 2018) (25) | COBRA |  |  |  |  |  | D | D |  |  |  | D | D |
| 26 | (Shi et al., 2019) (26) | NBSC |  |  |  |  |  |  |  |  | D |  |  | V |
| 27 | (Zhong et al., 2013) (27) | CSB |  |  |  |  |  | A | V |  | D |  | D | D |
| 28 | (Mehta et al., 2011) (28) | SOCRATIS | D | D | D | D | D |  | V |  |  |  | V | V |
| **Score:** V= very good; A = adequate; D = doubtful; I = inadequate; N= not applicable  **Abbreviation:** COBRA: Cognitive Complaints in Bipolar Disorder Rating Assessment; CSB: CogState Battery; NBSC: New Cognitive battery for patients with schizophrenia in China; pf: professional; pt: patients; SOCRATIS: Social Cognition Rating Tools in Indian Setting | | | | | | | | | | | | | | |

Title: Assessing cognition in people with severe mental disorders in low-and middle-income countries: a systematic review of assessment measures

Short title: Measures of cognition in severe mental disorders

Yohannes Gebreegziabhere^1, 2*^, Kassahun Habatmu^3^, Andualem Derese^2, 4^, Hetta Gouse^5^, Stephen M Lawrie^6^, Matteo Cella^7^, Atalay Alem^2^

^1^Department of Nursing, College of Health Sciences, Debre Berhan University, Debre Berhan, Ethiopia

^2^Department of Psychiatry, College of Health Sciences, Addis Ababa University, Addis Ababa, Ethiopia

^3^School of Psychology, College of Education and Behavioral Studies, Addis Ababa University, Addis Ababa, Ethiopia

^4^Department of Public Health, College of Health Sciences, Haremaya University, Harar, Ethiopia

^5^Department of Psychiatry and Mental Health, University of Cape Town, Cape Town, South Africa

^6^Department of Psychiatry, University of Edinburgh, Edinburgh, Scotland, United Kingdom

^7^Department of Psychology, Institute of Psychiatry, Psychology and Neuroscience, King's College London, London, England, United Kingdom

^*^Correspondence: Yohannes Gebreegziabhere Haile, Department of Nursing, Debre Berhan University, Debre Berhan, Ethiopia & Department of Psychiatry, College of Health Sciences, Addis Ababa University, Addis Ababa Ethiopia;

Phone number: +251 9 130 596 40;

Email: [yohannes36@gmail.com](mailto:yohannes36@gmail.com); or [yohannes36@dbu.edu.et](mailto:yohannes36@dbu.edu.et)

**Reference**

1. Araujo GE, Resende CB, Cardoso AC, Teixeira AL, Keefe RS, Salgado JV. Validity and reliability of the Brazilian Portuguese version of the BACS (Brief Assessment of Cognition in Schizophrenia). Clinics (Sao Paulo, Brazil). 2015;70:278-282.

2. Salgado JV, Carvalhaes CFR, Pires AM, Neves M, Cruz BF, Cardoso CS, Lauar H, Teixeira AL, Keefe RSE. Sensitivity and applicability of the Brazilian version of the Brief Assessment of Cognition in Schizophrenia (BACS). Dementia & neuropsychologia. 2007;1:260-265.

3. Mazhari S, Parvaresh N, Eslami Shahrbabaki M, Sadeghi MM, Nakhaee N, Keefe RS. Validation of the Persian version of the brief assessment of cognition in schizophrenia in patients with schizophrenia and healthy controls. Psychiatry and clinical neurosciences. 2014;68:160-166.

4. Muliady S, Malik K, Amir N, Kaligis F. Validity and Reliability of the Indonesian Version of Brief Assessment of Cognition in Schizophrenia (BACS-I). Journal of International Dental and Medical Research. 2019;12:263-267.

5. Abdullah H, Osman ZJ, Alwi MNM, Shah SA, Ibrahim N, Baharuddin A, Jaafar NRN, Said SM, Rahman HA, Bahari R. Reliability and Validity of the Malay Version of the Brief Assessment of Cognition in Schizophrenia (BACS): Preliminary Results. The European Journal of Social & Behavioural Sciences. 2013;5:920.

6. Azizian A, Yeghiyan M, Ishkhanyan B, Manukyan Y, Khandanyan L. Clinical validity of the Repeatable Battery for the Assessment of Neuropsychological Status among patients with schizophrenia in the Republic of Armenia. Archives of Clinical Neuropsychology. 2011;26:89-97.

7. Johnson I, Kebir O, Ben Azouz O, Dellagi L, Rabah Y, Tabbane K. The self-assessment scale of cognitive complaints in schizophrenia: a validation study in Tunisian population. BMC psychiatry. 2009;9:66.

8. Aydemir O, Cokmus FP, Akdeniz F, Suculluoglu Dikici D, Balikci K. Psychometric properties of the Turkish Versions of Perceived Deficit Questionnaire-Depression and British Columbia Cognitive Complaints Inventory. Anadolu Psikiyatri Dergisi. 2017;18:224-230.

9. Shi C, Wang G, Tian F, Han X, Sha S, Xing X, Yu X. Reliability and validity of Chinese version of perceived deficits questionnaire for depression in patients with MDD. Psychiatry research. 2017;252:319-324.

10. Bosgelmez S, Yildiz M, Yazici E, Inan E, Turgut C, Karabulut U, Kircali A, Tas HI, Yakisir SS, Cakir U, Sungur MZ. Reliability and validity of the Turkish version of cognitive assessment interview (CAI-TR). Klinik Psikofarmakoloji Bulteni / Bulletin of Clinical Psychopharmacology. 2015;25:365-380.

11. Changiz R, Razieh H, Norolah M. &lt;The&gt; utility of the Wisconsin card sorting test in differential diagnosis of cognitive disorders in Iranian psychiatric patients and healthy subjects. Iran J Psychiatry. 2011;6:99-105.

12. Dias FLDC, Teixeira AL, Guimaraes HC, Barbosa MT, Resende EDPF, Beato RG, Carmona KC, Caramelli P. Cognitive performance of community-dwelling oldest-old individuals with major depression: The Pieta study. International psychogeriatrics. 2017;29:1507-1513.

13. Fan HZ, Zhu JJ, Wang J, Cui JF, Chen N, Yao J, Tan SP, Duan JH, Pang HT, Zou YZ. Four-Subtest Index-based Short Form of WAIS-IV: Psychometric Properties and Clinical Utility. Archives of clinical neuropsychology : the official journal of the National Academy of Neuropsychologists. 2019;34:81-88.

14. Fonseca AO, Berberian AA, de Meneses-Gaya C, Gadelha A, Vicente MDO, Nuechterlein KH, Bressan RA, Lacerda ALT. The Brazilian standardization of the MATRICS consensus cognitive battery (MCCB): Psychometric study. Schizophrenia research. 2017;185:148-153.

15. Mazhari S, Ghafaree-Nejad AR, Soleymani-Zade S, Keefe RSE. Validation of the Persian version of the Schizophrenia Cognition Rating Scale (SCoRS) in patients with schizophrenia. Asian journal of psychiatry. 2017;27:12-15.

16. Negrão J, Akiba HT, Lederman VRG, Dias ÁM. Faux Pas Test in schizophrenic patients. J bras psiquiatr. 2016;65:17-21.

17. Sanvicente-Vieira B, Brietzke E, Grassi-Oliveira R. Translation and adaptation of Theory of Mind tasks into Brazilian portuguese. Trends psychiatry psychother (Impr). 2012;34:178-185.

18. Morozova A, Garakh Z, Bendova M, Zaytseva Y. Comparative Analysis of Theory of Mind Tests in First Episode Psychosis Patients. Psychiatria Danubina. 2017;29:285-288.

19. Pieters HC, Sieberhagen JJ. Evaluation of two shortened forms of the SAWAIS with three diagnostic groups. Journal of clinical psychology. 1986;42:809-815.

20. Ruzita J, Zahiruddin O, Kamarul Imran M, Muhammad Najib Muhammad A. Validation of The Malay Version of Auditory Verbal Learning Test (MVAVLT) Among Schizophrenia Patients In Hospital Universiti Sains Malaysia (HUSM), Malaysia. ASEAN Journal of Psychiatry. 2009:54-74.

21. Tuncay N, Kayserili G, Eser E, Zorlu Y, Akdede BB, Yener G. Validation and reliability of the frontal assesment battery (FAB) in Turkish. Journal of Neurological Sciences. 2013;30:502-514.

22. Gulec H, Kavakci O, Gulec MY, Kucukali CI, Citak S. The validity and reliability of Turkish version of Frontal Assessment Battery in patients with schizophrenia. Neurology Psychiatry and Brain Research. 2008;14:165-168.

23. Lima FM, Cardoso TA, Serafim SD, Martins DS, Solé B, Martínez-Arán A, Vieta E, Rosa AR. Validity and reliability of the Cognitive Complaints in Bipolar Disorder Rating Assessment (COBRA) in Brazilian bipolar patients. Trends psychiatry psychother (Impr). 2018;40:170-178.

24. Xiao L, Lin X, Wang Q, Lu D, Tang S. Adaptation and validation of the "cognitive complaints in bipolar disorder rating assessment" (COBRA) in Chinese bipolar patients. Journal of affective disorders. 2015;173:226-231.

25. Yoldi-Negrete M, Fresan-Orellana A, Martinez-Camarillo S, Ortega-Ortiz H, Juarez Garcia FL, Castaneda-Franco M, Tirado-Duran E, Becerra-Palars C. Psychometric properties and cross-cultural comparison of the cognitive complaints in bipolar disorder rating assessment (COBRA) in Mexican patients with bipolar disorder. Psychiatry research. 2018;269:536-541.

26. Shi C, Kang L, Yao S, Ma Y, Li T, Liang Y, Cheng Z, Xu Y, Shi J, Xu X. What is the optimal neuropsychological test battery for schizophrenia in China? Schizophrenia research. 2019;208:317-323.

27. Zhong N, Jiang H, Wu J, Chen H, Lin S, Zhao Y, Du J, Ma X, Chen C, Gao C. Reliability and validity of the CogState battery Chinese language version in schizophrenia. PloS one. 2013;8:e74258.

28. Mehta UM, Thirthalli J, Naveen Kumar C, Mahadevaiah M, Rao K, Subbakrishna DK, Gangadhar BN, Keshavan MS. Validation of Social Cognition Rating Tools in Indian Setting (SOCRATIS): A new test-battery to assess social cognition. Asian journal of psychiatry. 2011;4:203-209.
